# Phylogeny, character evolution and spatiotemporal diversification of the species-rich and world-wide distributed tribe Rubieae (Rubiaceae)

**DOI:** 10.1371/journal.pone.0207615

**Published:** 2018-12-05

**Authors:** Friedrich Ehrendorfer, Michael H. J. Barfuss, Jean-Francois Manen, Gerald M. Schneeweiss

**Affiliations:** 1 Department of Botany and Biodiversity Research, University of Vienna, Vienna, Austria; 2 Laboratoire de Systématique Végétale et Biodiversité, University of Geneva, Geneva, Switzerland; Georg-August-Universitat Gottingen, GERMANY

## Abstract

The Rubiaceae tribe Rubieae has a world-wide distribution with up to 1,000 species. These collectively exhibit an enormous ecological and morphological diversity, making Rubieae an excellent group for macro- and microevolutionary studies. Previous molecular phylogenetic analyses used only a limited sampling within the tribe or missed lineages crucial for understanding character evolution in this group. Here, we analyze sequences from two plastid spacer regions as well as morphological and biogeographic data from an extensive and evenly distributed sampling to establish a sound phylogenetic framework. This framework serves as a basis for our investigation of the evolution of important morphological characters and the biogeographic history of the Rubieae. The tribe includes three major clades, the Kelloggiinae Clade (*Kelloggia*), the Rubiinae Clade (*Didymaea*, *Rubia*) and the most species-rich Galiinae Clade (*Asperula*, *Callipeltis*, *Crucianella*, *Cruciata*, *Galium*, *Mericarpaea*, *Phuopsis*, *Sherardia*, *Valantia*). Within the Galiinae Clade, the largest genera *Galium* and *Asperula* are para- and polyphyletic, respectively. Smaller clades, however, usually correspond to currently recognized taxa (small genera or sections within genera), which may be used as starting points for a refined classification in this clade. Life-form (perennial versus annual), flower shape (long versus short corolla tube) and fruit characters (dry versus fleshy, with or without uncinate hairs) are highly homoplasious and have changed multiple times independently. Inference on the evolution of leaf whorls, a characteristic feature of the tribe, is sensitive to model choice. Multi-parted leaf whorls appear to have originated from opposite leaves with two small interpetiolar stipules that are subsequently enlarged and increased in number. Early diversification of Rubieae probably started during the Miocene in western Eurasia. Disjunctions between the Old and the New World possibly are due to connections via a North Atlantic land bridge. Diversification of the Galiineae Clade started later in the Miocene, probably in the Mediterranean, from where lineages reached, often multiple times, Africa, eastern Asia and further on the Americas and Australia.

## Introduction

Rubiaceae is the fourth-largest family of angiosperms and includes about 12000, mostly woody and tropical species [1]. Within this family, the predominantly herbaceous tribe Rubieae, comprising up to 1,000 species in about twelve genera [2], has achieved a world-wide distribution and an enormous ecological diversity with species in tropical habitats, Mediterranean bushlands, temperate deciduous forests or arctic and alpine tundra. This is paralleled by a considerable morphological diversity, e.g., with respect to life form (perennials, annuals), growth form (shrubs, vines, herbs), leaves and stipules, inflorescences, flower and fruit morphology and corresponding changes in pollination (e.g., tubular or rotate corollas for different visitors) and dispersal ecology (e.g., dry mericarps with uncinate hairs as means for epizoochory or fleshy fruits as means for endozoochory). Some species have become globally distributed weeds, e.g., *Galium aparine* [3]. Hence, Rubieae is an excellent group for studies on macroeveolution (e.g., large-scale biogeographic relationships, explosive radiations) and microevolution (e.g., polyploidy, invasiveness).

Rubieae has been subject to a number of molecular phylogenetic studies. Many of those addressed circumscription, position and elementary cladogenesis within the tribe [4–9], usually with limited sampling within the tribe. Soza and Olmstead [10–11] achieved a more extensive survey of Rubieae, but as they focused on New World lineages, they missed some lineages crucial for understanding character evolution in this group (e.g., *Galium paradoxum* and particular sections). A preliminary report on phylogenetic relationships within Rubieae using an extended taxon sampling was given by Ehrendorfer and Barfuss [12]. Recently, Yang et al. [13] addressed relationships within Rubieae using a broad sampling with an expanded focus on species occurring in China.

A prominent feature of Rubieae is the evolution of leaf whorls with four or more elements. Although there is ambiguity with respect to the interpretation of the Rubieae leaf whorls (summarized in [14]), comparison with the phyllotaxis in other Rubiaceae suggests that these whorls most likely evolved from opposite leaves with two interpetiolar stipules via enlargement and increase in number of stipules. This plausible evolutionary sequence is supported by some statistical character state reconstructions [13], but is in obvious conflict with others [10], which suggest leaf/stipule whorls with at least six elements as ancestral in Rubieae. Further character changes of interest, which often have been used for taxonomic purposes, include life-form (perennials versus annuals), corolla shape (trumpet-like versus rotate, historically used for the separation of *Asperula* and *Galium*), pollen type (used to distinguish Rubieae from other tribes [8,9]) as well as fruit structure (dry versus fleshy). By altering, for instance, pollination or dispersal, these changes may be responsible for the success of Rubieae species in many different habitats including, especially for weedy species, also man-made ones. Testing such hypotheses, however, requires a solid understanding of character evolution in the entire tribe, which is lacking so far.

Using an extensive and evenly distributed sampling across Rubieae, we aim to establish a well-supported phylogenetic framework as a basis for reconstruction of the evolution of growth form, phyllotaxis, flower and fruit morphology and for inferring the tribe’s biogeographic history employing, compared to previous studies [10], a much refined set of geographic areas. To this end, we analyze sequences from two plastid spacer regions, *atpB-rbcL* and *rpl32*-*trnL*, as well as morphological and biogeographic data using maximum parsimony and Bayesian methods. Specifically, we aim (i) to phylogenetically place so far unstudied Rubieae (such as *Mericarpaea*), thus providing a refined framework to evaluate discrepancies between phylogenetic relationships and the current taxonomy of the tribe, (ii) to test existing hypotheses (e.g., [10,13]) on the evolution of leaf whorls, and (iii) to develop a biogeographic scenario for Rubieae.

## Materials and methods

### Ethics statement

The investigated taxa are neither endangered nor protected. All material was collected on public land, where no special permissions are required.

### DNA sequencing

Total genomic DNA was extracted from silica gel dried leaf tissue or from herbarium specimens (S1 Table) using the 2× CTAB protocol described by Doyle and Doyle [15] modified for microcentrifuge tubes. The DNeasy Plant Mini Kit (QIAGEN, Hilden, Germany) was sometimes used for difficult material according to the manufacturer’s instructions.

In addition to the plastid DNA *atpB*-*rbcL* spacer from the large single-copy region (LSC) used previously [5,7], the highly variable *rpl32*-*trnL* spacer from the small single-copy region (SSC) was sequenced [16,17]. Apart from several *atpB*-*rbcL* spacer sequences downloaded from GenBank, all sequences used in this study were newly obtained. Primers used were Oligo 2 (5'-GAA GTA GTA GGA TTG ATT CTC-3') and Oligo 5 (5'-TAC AGT TGT CCA TGT ACC AG-3') for *atpB*-*rbcL* [5] and rpL32-F (5'-CAG TTC CAA AAA AAC GTA CTT C-3') and trnL^(UAG)^ (5'-CTG CTT CCT AAG AGC AGC GT-3') for *rpl32*-*trnL* [16].

In Geneva, the *atpB*-*rbcL* spacer was amplified and sequenced (mostly manually by the Sanger sequencing method) as described in Manen et al. [5] and Natali et al. [7]. In Vienna, the *atpB*-*rbcL* and *rpl32*-*trnL* spacers were sequenced as follows: The PCR reaction mix contained in a final reaction volume (usually 10 μl): (1) 5 μl ThermoPrime 2× ReddyMix PCR Master Mix with 1.5 mM MgCl_2_ (Thermo Fisher Scientific, Vienna, Austria); (2) 1 μl of 3.2 μM each primer (Sigma-Aldrich, Vienna, Austria; SA); (3) 0.1 μl of 20 mg/ml BSA (Thermo Fisher Scientific); (4) 1.9 μl of 1 M Trehalose (Sigma-Aldrich, Vienna, Austria); and (5) 1 μl of diluted DNA template. The PCR reactions were performed in an Eppendorf Mastercycler (Eppendorf, Vienna, Austria) with modifications of standard cycling conditions recommended by Su et. al. [18]: 1 cycle with 95°C for 2 min; 35 cycles each with 95°C for 25 s, 48°C for 35 s, 68°C for 1 min; 1 cycle with 72°C for 5 min; and a final hold at 15°C. PCR products were purified with a 1:2 mixture of Exonuclease I (20 U/μl; Thermo Fisher Scientific) and FastAP Thermosensitive Alkaline Phosphatase (1 U/μl; Thermo Fisher Scientific) according to Werle et al. [19]. We added 1 μl of the enzyme mixture to each 9 μl PCR reaction (after verifying 1 μl on an agarose gel) and incubated at 37°C for 45 min, followed by a deactivation of the enzymes at 85°C for 15 min.

Cycle sequencing reactions were performed on a 96-Well GeneAmp PCR System 9700 (Thermo Fisher Scientific) according to the BigDye Terminator v3.1 Cycle Sequencing Kit (Thermo Fisher Scientific) using PCR primers. We used slightly modified PCR conditions: 1 cycle with 96°C for 1 min; 35 cycles each with 96°C for 10 s, 50°C for 5 s; 60°C for 3 min; and a final hold at 4°C. Reaction components were: 0.4 μl of BigDye Terminator v3.1, 1 μl of 3.2 μM primer, 1.8 μl of 5× sequencing buffer (Thermo Fisher Scientific), 2 μl of 1 M Trehalose, 2 (or 4) μl of purified PCR product, and 2.8 (or 0.8) μl of PCR-grade water. Sephadex-cleaned products were run on a 3130xL Genetic Analyzer or 3730 DNA Analyzer (Thermo Fisher Scientific) following manufacturer’s instructions.

Sequences were assembled and edited using SeqMan Pro (Lasergene 8.1, DNASTAR), and the consensus was exported in fasta format and deposited in GenBank (S1 Table). DNA sequence alignments were generated manually in the program BioEdit 7.2.5 [20,21] for each investigated plastid locus. Because of the linked nature of plastid sequences, DNA sequences were concatenated (loci not available for some samples were included as missing data; S1 Table), henceforth referred to as complete data set. A second data set was generated after preliminary phylogenetic analyses by eliminating duplicate samples of monophyletic species, henceforth referred to as reduced data set.

### Phylogenetic analyses and ancestral character state reconstruction

Phylogenetic reconstructions were obtained using maximum parsimony (MP) on the complete data set (comprising 252 accessions from 165 species, one with two subspecies, of Rubieae plus one accession each from two outgroups, *Theligonum* and *Putoria*, chosen based on previous phylogenetic evidence [22]) as well as Bayesian inference (BI) on the reduced data set (comprising 183 accessions including the outgroups). The MP analyses were performed with PAUP* 4.0a149 [23,24] using 1000 replicates of heuristic search with random addition of sequences (five trees held at each step) and subsequent TBR branch swapping (steepest descent option not in effect, MULTREES option in effect, branches collapsed if maximum branch length is zero, and saving no more than 50 trees in each replicate). All analyses were performed with DNA sequence characters treated as independent, unordered and equally weighted and with gaps treated as missing data. Parsimony branch support was calculated with the bootstrap using 1000 pseudo-replicates, which were performed in the same way as the MP analyses, but using 10 replicates of heuristic search with random sequence addition and subsequent TBR branch swapping (saving no more than 10 trees in each replicate).

Bayesian phylogenetic analyses were conducted on the reduced data set using BEAST 1.8.x [25,26]. Model-fit of nucleotide substitution models was assessed via the Akaike Information Criterion (AIC) as implemented in MODELTEST v3.8 [27]. As for both sequence markers models of similar complexity have been identified (GTR+I+G for the *atpB-rbcL* spacer and TVM+I+G for the *rpl32-trnL* spacer, the models having 10 and 9 free parameters, respectively) and because both markers are linked intergenic spacers, the data set was not partitioned and a single GTR+I+G model was used. As priors for the rate parameters as well as the shape parameter of the gamma distribution and the proportion of invariable sites we used normal distributions constructed to have the highest probability density at the value for the respective parameter estimated for the best supported model and standard deviations of at least one sixth of the mean. As tree prior we used a speciation model following a Yule process, the prior distribution of this process’ parameter being modelled as a normal distribution with mean 0.6 and standard deviation of 0.5. Analyses consisted of two runs for 2×10^8^ generations each with sampling every 10,000^th^ generation; as both runs converged on the same stationary distribution (determined using Tracer 1.4, available from http://tree.bio.ed.ac.uk/software/tracer/) and effective sample size (ESS) values safely exceeded 1000, they were combined after removal of the first 10% of sampled generations as burn-in, resulting in a total of 36,000 sampling points.

Molecular clock models (strict clock or uncorrelated relaxed clock with rates drawn from a lognormal distribution) were selected based on marginal likelihood estimations via path sampling (PS) and stepping-stone (SS) sampling [28]. After an initial phase of 2×10^8^ generations, MCMC samples (chain length of 5×10^6^ generations) were drawn from a series of 100 power posteriors (differing in their power determined by evenly spaced quantiles of a Beta (0.3, 1.0) distribution [29]) along the path from the prior to the un-normalized posterior [28]. This was done twice, and the results from these independent runs were combined.

Age priors were applied on the two nodes pertaining (i) to the split between the North American and the East Asian *Kelloggia* species and (ii) to the stem node of Rubieae (i.e., the clade of Rubieae plus *Theligonum*); as each of these clades had high support in unconstrained analyses (data not shown), they were constrained to be monophyletic for the final analyses. The split between the two *Kelloggia* species has been estimated to be 5.4 ± 3.2 million years ago (mya; [30]), which was realized with a lognormal distribution with (in real space) a mean of 5.5 mya and a standard deviation of 0.2 mya. The stem node age of Rubieae has been estimated as 28.6 mya (20.2–37.6 mya; [31]), which was realized with a lognormal distribution with (in real space) a mean of 29 mya and a standard deviation of 0.3 mya.

A second set of BEAST analyses was conducted to explore the evolution of several important morphological traits and to provide a biogeographic scenario for the tribe. The following morphological characters have been included (S1 Table): (1) life form (perennial, annual); (2) leaves and stipules (decussate leaves with two small median stipules; decussate leaves with enlarged stipules; leaf whorl with mostly four equal-sized elements; leaf whorls with at least six elements); (3) corolla tube (long, i.e., >1.5 mm; short, i.e., <1.5 mm); (4) pollen type (tricolpate, polycolpate, polyporate); (5) fruit type (dry, fleshy); (6) fruit indumentum (without uncinate hairs, with uncinate hairs). Character evolution was described using asymmetric continuous-time Markov chain (CTMC) models (i.e., reverse rates are allowed to differ from forward rates) with uniform rates across branches (i.e., a strict clock model). For the number of leaf elements, both a model of unordered character state change and a model of ordered character state change were used. In the latter, character states are ordered according to an increase in number and size of stipular elements (see above) in agreement with previous hypotheses [13]. Priors on the trait rates were modelled as gamma distributions with both shape and scale 1.0, those on the clock rates were modelled as exponential distributions with mean 1.0. Geography was coded as nine discrete non-overlapping regions based on biogeographic considerations (presence of endemic Rubieae taxa): North America, Central America, South America, Mediterranean, Africa South of the Sahara, western Eurasia (mostly Europe), southwestern Asia, eastern Eurasia (mostly East Asia), Australia. Biogeographic history was modelled using a symmetric CTMC model [32]. We used Bayesian stochastic search variable selection (BSSVS) to reduce the number of nonzero rates. To this end, a truncated Poisson prior with a mean of 0.693 (i.e., *ln*2) and an offset corresponding to the number of rates necessary to minimally connect all regions (i.e., number of regions minus 1) was used, which puts 50% prior probability on the minimal rate configuration. We used equal expectations for all rates, i.e., the prior on the diffusion rates is not informed by the geographic distances among geographic units. As results from the non-reversible model (the diffusion rate in one direction can differ from that in the reverse direction) are more sensitive to the BSSVS priors and as this model tends to support disproportionally more rates than the reversible model [33], we restrict ourselves to the more stable reversible model.

Ancestral character state reconstruction was additionally done under maximum parsimony using Mesquite 3.04 [34]. Reconstructions were made on 6,000 posterior trees from the analyses with molecular data only (using “trace character over trees”) and were plotted on a 50% majority rule consensus tree (data matrix including trees available as S2 Appendix). For the number of leaf elements, both unordered and ordered parsimony were used. For some nodes, reconstruction uncertainty was quantified using the index of reconstruction precision, *RP*_*n*_, defined by McCann et al. [35]. This index ranges from 0 (minimum precision and maximum uncertainty) to 1 (maximum precision and no uncertainty).

The BEAST xml input file with the combined data (i.e., including DNA sequences, trait data and biogeographic assignments) is available as S1 Appendix.

## Results

### Phylogeny

**Fig 1:**
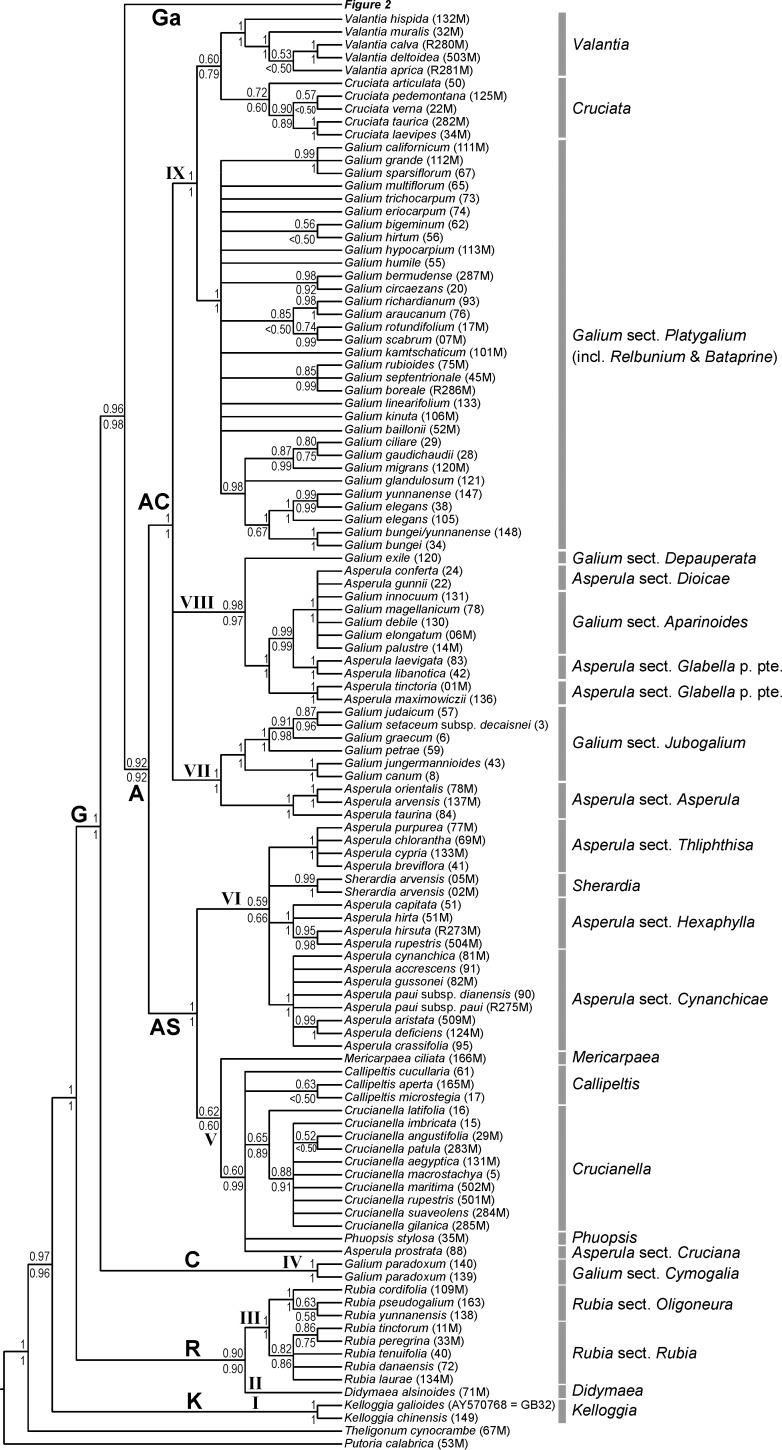
Phylogenetic relationships of Rubieae inferred from Bayesian analysis with a relaxed clock of molecular data from the reduced data set. Shown is the 50% majority rule consensus tree (for details of the Galium Clade see Fig 2); values above branches are posterior probabilities of at least 0.5 from a Bayesian analysis with molecular data only and values below branches are posterior probabilities of at least 0.5 from a Bayesian analysis with combined data (molecular data, trait data, biogeographical data). Roman numerals indicate clade designations used by Ehrendorfer and Barfuss [12]. Abbreviations of major clades: **A**, Asperula Clade; **AC**, Asperula-Cruciata Clade; **AS**, Asperula-Sherardia Clade; **C**, Cymogalia Clade; **G**, Galiinae Clade; **Ga**, Galium Clade; **K**, Kelloggiinae Clade; **R**, Rubiinae Clade.

**Fig 2:**
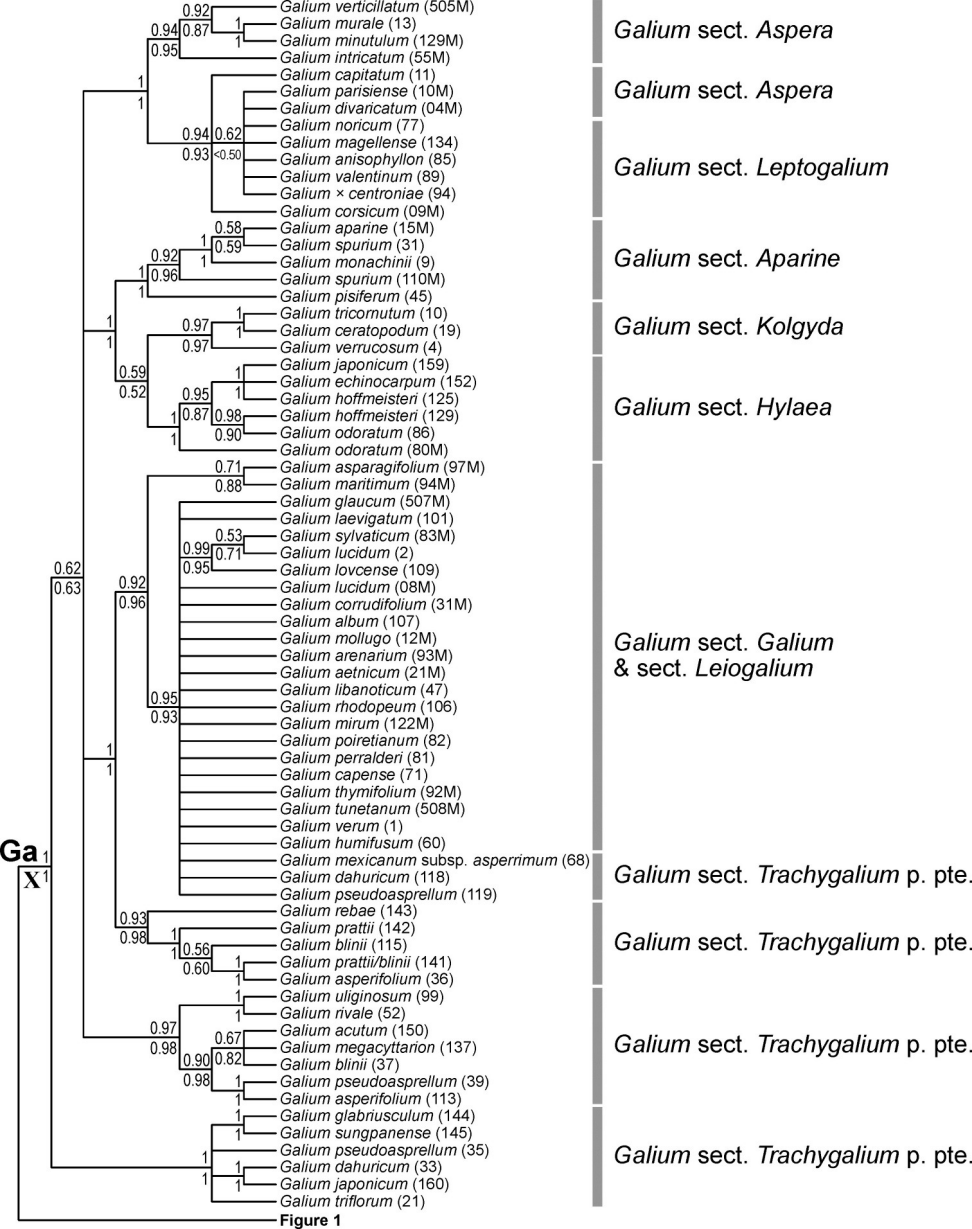
Phylogenetic relationships of Rubieae inferred from Bayesian analysis with a relaxed clock of molecular data from the reduced data set: The Galium Clade. Shown is the 50% majority rule consensus tree; values above branches are posterior probabilities of at least 0.5 from a Bayesian analysis with molecular data only and values below branches are posterior probabilities of at least 0.5 from a Bayesian analysis with combined data (molecular data, trait data, biogeographical data). The Roman numeral indicates clade designation used by Ehrendorfer and Barfuss [12]. Abbreviations of the major clade: **Ga**, Galium Clade.

Phylogenetic relationships inferred from maximum parsimony and Bayesian methods are largely congruent and without any strongly supported incongruences (Figs 1 and 2 and S1). Therefore, in the following we will focus on the results from the Bayesian analysis (s 1–2; for maximum parsimony bootstrap support values see S1 Fig). Bayes Factors based on marginal likelihoods estimated via stepping stone or path sampling support a relaxed clock model over a strict clock model, both in the data set including DNA sequences only (logBF_RC-SC_ 41.01 and 41.86, respectively) as well as in the combined data set additionally including morphological traits and geographic data (logBF_RC-SC_ 44.45 and 43.72, respectively).

*Galium*, the most species-rich genus of the tribe, is paraphyletic, forming the well-supported Galiinae Clade together with nested genera, such as *Asperula* or *Crucianella* (clade **G** in Fig 1; maximum parsimony bootstrap BS / posterior probability molecular data only PP^m^ / posterior probability combined molecular and trait data PP^c^ 84/1/1; corresponds to the “superclade *Galium* s.l.” as defined by Ehrendorfer and Barfuss [12]; see Table 1 for correspondence of clade designations between this and previous studies [10,12,13]). Sister group (BS/PP^m^/PP^c^ 100/1/1) to the Galiinae Clade is the Rubiinae Clade (clade **R** in Fig 1; BS/PP^m^/PP^c^ <50/0.90/0.90). The Rubiinae Clade includes *Didymaea* (only one sample included, hence no support values) and *Rubia* (BS/PP^m^/PP^c^ 78/1/1). Within *Rubia*, two subclades are distinguishable corresponding to the mostly Mediterranean and southwestern Asian species of *Rubia* sect. *Rubia* (including *R*. sect. *Campylanthera*; BS/PP^m^/PP^c^ <50/0.82/0.86) and the eastern Eurasian centered species of *R*. sect. *Oligoneura* (BS/PP^m^/PP^c^ 68/1/1). The Kelloggiinae Clade (clade **K** in Fig 1; BS/PP^m^/PP^c^ 72/1/1), sister to the rest of the Rubieae (BS/PP^m^/PP^c^ 88/0.97/0.96), consists only of *Kelloggia*, with two species showing an East Asian–western North-American disjunction.

**Table 1:** Designations of major clades of Rubieae identified here and in previous studies.

This study	Soza and Olmstead [10]	Ehrendorfer and Barfuss [12]	Yang et al. [13]
Kelloggiinae Clade (**K**)	n. d. (part of outgroup)	Clade I	n. d. (part of outgroup)
Rubiinae Clade (**R**)	Clades I+II	Clades II+III	n. d. (part of outgroup)
n. d. (*Didymaea*)	Clade I	Clade II	n. d. (part of outgroup)
n. d. (*Rubia*)	Clade II	Clade III	n. d. (part of outgroup)
Galiinae Clade (**G**)	Clades III–VII	Clades IV–X	Clades I–III
Cymogalia Clade (**C**)	n. i.	Clade IV	Clade I
Asperula Clade (**A**)	Clades IV–VII	Clades V–IX	Clade III
Asperula-Sherardia Clade (**AS**)	Clade IV	Clades V+VI	Clade III-a
n. d.	Clade IV-F	Clade V	n. d.
n. d.	Clade IV-E	Clade VI	n. d.
Asperula-Cruciata Clade (**AC**)	Clades V–VII	Clades VII–IX	Clades III-b–III-e
n. d.	Clade VI	Clade VII	Clade III-c
n. d.	Clade V	Clade VIII	Clade III-b
n. d.	Clade VII	Clade IX	Clades III-d + III-e
Galium Clade (**Ga**)	Clade III	Clade X	Clade II

Abbreviations: n. d.: not denominated; n. i.: not included

Within the Galiinae Clade, the Cymogalia Clade (clade **C** in Fig 1; BS/PP^m^/PP^c^ 100/1/1), corresponding to the monotypic *Galium* sect. *Cymogalia* ser. *Paradoxa*, is inferred as sister (BS/PP^m^/PP^c^ 64/0.96/0.98) to all remaining taxa (i.e., clade **Ga** plus clade **A**). These remaining taxa are grouped into the Asperula Clade (clade **A** in Fig 1; BS/PP^m^/PP^c^ <50/0.92/0.92) and the Galium Clade (clade **Ga** in Figs 1 and 2; BS/PP^m^/PP^c^ 55/1/1). Whereas the Galium Clade contains exclusively species of *Galium*, the Asperula Clade includes, in addition to several members of *Galium*, the genera *Asperula*, *Callipeltis*, *Crucianella*, *Cruciata*, *Mericarpaea*, *Phuopsis*, *Sherardia*, and *Valantia*. Leaving the monotypic *Mericarpaea*, *Phuopsis*, and *Sherardia* aside, the remaining genera restricted to the Asperula Clade are either monophyletic (*Crucianella*, BS/PP^m^/PP^c^ <50/0.65/0.89; *Cruciata*, BS/PP^m^/PP^c^ <50/0.72/0.60; *Valantia*, BS/PP^m^/PP^c^ 85/1/1), unresolved (*Callipeltis* is part of a polytomy, except in the Bayesian analysis with traits included, where it receives PP 0.99) or polyphyletic (*Asperula*).

The Asperula Clade falls into two clades (each BS/PP^m^/PP^c^ ≤50/1/1), the Asperula-Sherardia Clade (clade **AS** in Fig 1) and the Asperula-Cruciata Clade (clade **AC** in Fig 1). The Asperula-Sherardia Clade is devoid of any *Galium* species, but contains several sections of *Asperula* and the genera *Callipeltis*, *Crucianella*, *Mericarpaea*, *Phuopsis*, and *Sherardia*. The Asperula-Sheradia Clade is divided into two clades: one comprises *Callipeltis*, *Crucianella*, *Mericarpaea*, *Phuopsis*, and *Asperula* sect. *Cruciana* (clade V in Fig 1; BS/PP^m^/PP^c^ <50/0.62/0.60), the other *Sherardia* and *Asperula* sects. *Cynanchicae*, *Hexaphylla*, and *Thliphthisa* (clade VI in Fig 1; BS/PP^m^/PP^c^ <50/0.59/0.60). Distinguishable smaller subclades correspond to traditionally recognized entities (sections of *Asperula* or genera, except *Callipeltis*, which comprises two lineages as part of a polytomy) and are mostly well supported (exceptions being *Crucianella* and *Callipeltis* p. pte. with BS/PP^m^/PP^c^ <50/0.65/0.89 and BS/PP^m^/PP^c^ 94/0.63/<0.50, respectively). Relationships among these small clades remain unclear due to lack of resolution or insufficient support.

The Asperula-Cruciata Clade (clade **AC** in Fig 1) comprises three well-supported clades with unclear relationships to each other. In the first clade (clade VII in Fig 1; BS/PP^m^/PP^c^ 94/1/1), *Asperula* sect. *Asperula* (BS/PP^m^/PP^c^ 73/1/1) and *Galium* sect. *Jubogalium* (BS/PP^m^/PP^c^ 98/1/1) are united as sister-groups. The second clade (clade VIII in Fig 1; BS/PP^m^/PP^c^ 69/0.98/0.97) contains several *Asperula* and *Galium* lineages. Specifically, *Asperula* sect. *Dioicae* and *Galium* sect. *Aparinoides* group together (BS/PP^m^/PP^c^ 88/1/1) with unresolved internal relationships. Their sister-group (BS/PP^m^/PP^c^ 91/0.99/0.99) is a clade (BS/PP^m^/PP^c^ 100/1/1) of two members of *Asperula* sect. *Glabella*, and the subsequent sister-group (BS/PP^m^/PP^c^ 92/1/1) is a clade (BS/PP^m^/PP^c^ 63/1/1) that contains the remaining species of *Asperula* sect. *Glabella*. The sister-group to the all other species of clade VIII is the single species of *Galium* sect. *Depauperata*. The third clade (clade IX in Fig 1; BS/PP^m^/PP^c^ 66/1/1) comprises the genera *Valantia* (BS/PP^m^/PP^c^ 85/1/1) and *Cruciata* (BS/PP^m^/PP^c^ <50/0.72/0.60), inferred as weakly supported sister groups (BS/PP^m^/PP^c^ <50/0.60/0.79), together with the internally poorly resolved clade (BS/PP^m^/PP^c^ <50/1/1) of *Galium* sect. *Platygalium* (including the nested former genera *Bataprine* and *Relbunium*).

In the Galium Clade (clade **Ga** in Figs 1 and 2), several subclades are found. Some of these corresponded to traditionally recognized sections of *Galium*, including the perennial sect. *Hylaea* (BS/PP^m^/PP^c^ 84/1/1) and the annual sects. *Aparine* (BS/PP^m^/PP^c^ 78/1/1) and *Kolgyda* (BS/PP^m^/PP^c^ 50/0.97/0.97), which all three together form one clade (BS/PP^m^/PP^c^ <50/1/1). Species of the perennial *G*. sect. *Leptogalium* together with a few species of the annual *G*. sect. *Aspera* form a clade (BS/PP^m^/PP^c^ <50/0.94/0.93), which is sister (BS/PP^m^/PP^c^ 62/1/1) to a clade containing the remaining species of *G*. sect. *Aspera* (BS/PP^m^/PP^c^ 51/0.94/0.95). The perennial species of *G*. sects. *Galium* and *Leiogalium* form, together with a few accessions from *G*. sect. *Trachygalium*, a clade (BS/PP^m^/PP^c^ 58/0.92/0.96) that is sister (BS/PP^m^/PP^c^ 87/1/1) to a clade containing some East Asian species of *G*. sect. *Trachygalium* (BS/PP^m^/PP^c^ 66/0.93/0.98). The remaining species of the heterogenous *G*. sect. *Trachygalium* are distributed over two clades (BS/PP^m^/PP^c^ <50/0.97/0.98 and 98/1/1, respectively). Relationships along the backbone of the Galium Clade remain unresolved or are insufficiently supported.

### Character evolution

**Fig 3:**
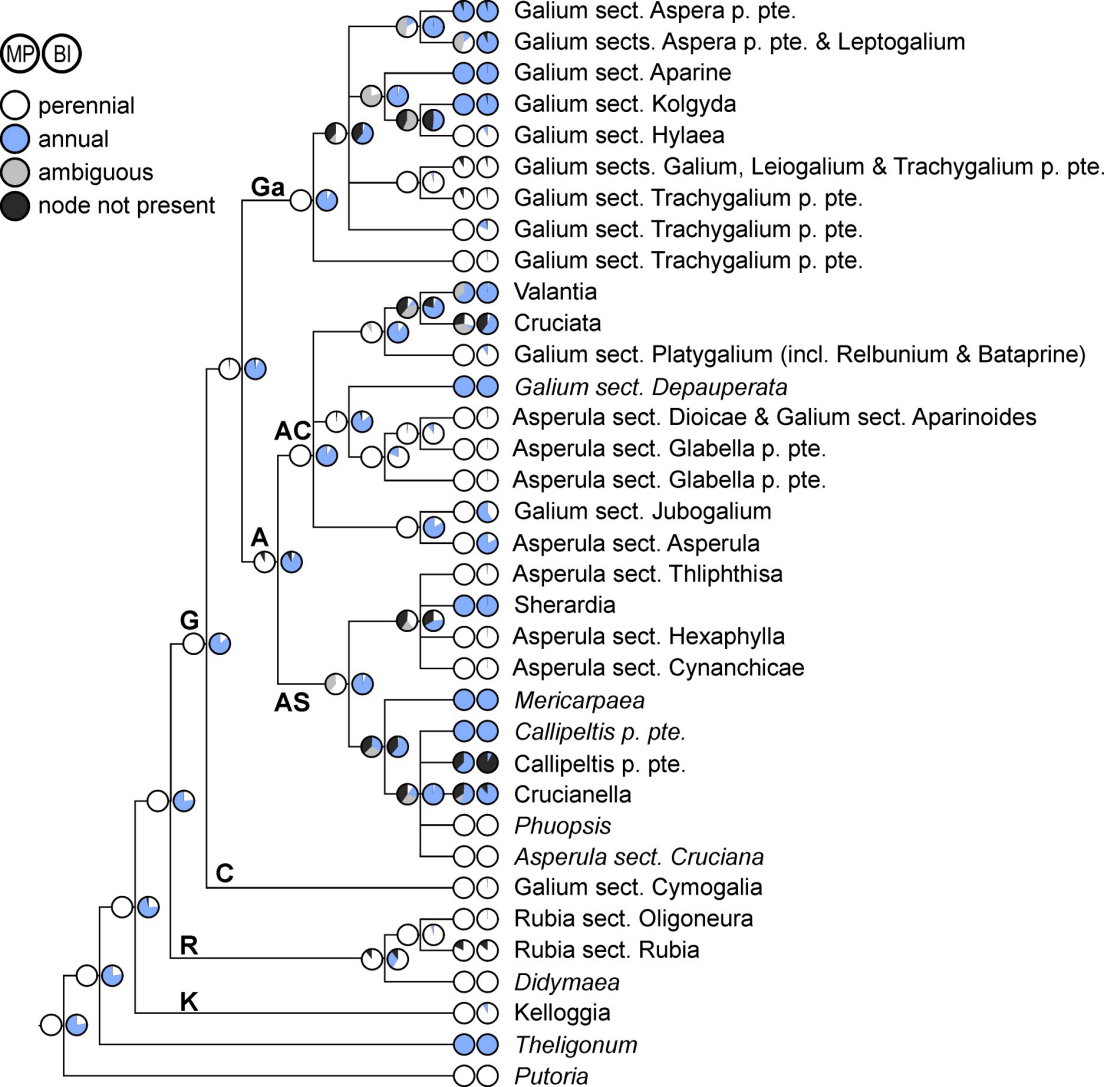
Evolution of life form (perennial/annual) in Rubieae. Ancestral character states reconstructed over a set of posterior trees using maximum parsimony (left pie charts) and Bayesian inference (right pie charts) are shown on a simplified majority rule consensus tree; therefore, terminal pie charts often show reconstructed character states at the crown node of small terminal clades (indicated by names in normal print) and only rarely the character states of a single accession (indicated by names in italics). Abbreviations of major clades: **A**, Asperula Clade; **AC**, Asperula-Cruciata Clade; **AS**, Asperula-Sherardia Clade; **C**, Cymogalia Clade; **G**, Galiinae Clade; **Ga**, Galium Clade; **K**, Kelloggiinae Clade; **R**, Rubiinae Clade.

**Fig 4:**
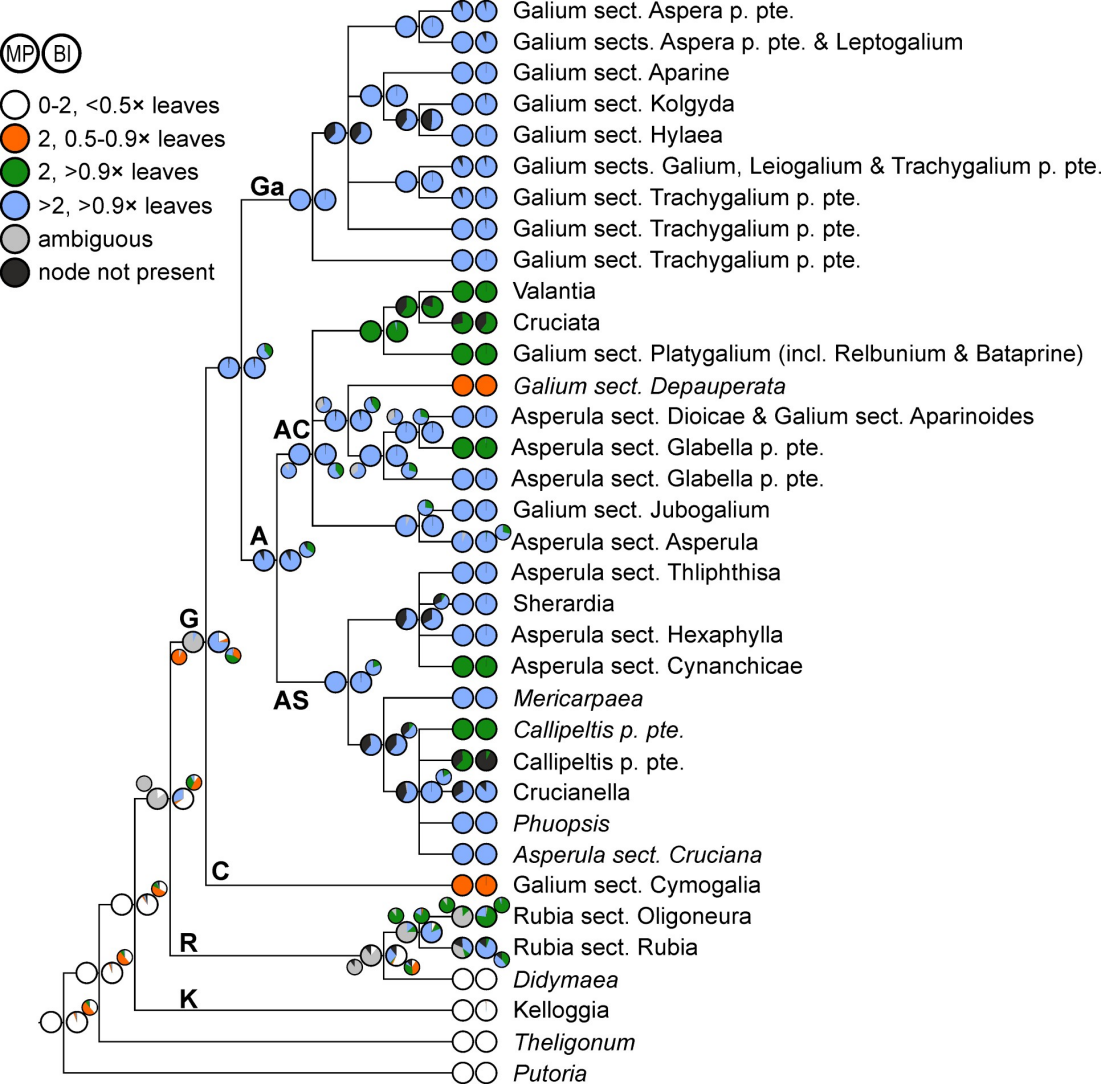
Evolution of stipules/stipular elements with respect to their number (0 to >2) and size (relative to the true leaves) in Rubieae. Ancestral character states reconstructed over a set of posterior trees using maximum parsimony (left pie charts) and Bayesian inference (right pie charts) are shown on a simplified majority rule consensus tree; therefore, terminal pie charts often show reconstructed character states at the crown node of small terminal clades (indicated by names in normal print) and only rarely the character states of a single accession (indicated by names in italics). Smaller pie charts indicate reconstructions under a model of ordered character state evolution; these are shown only in cases where reconstructions deviate considerably from those using unordered character states (see text for details). Abbreviations of major clades: **A**, Asperula Clade; **AC**, Asperula-Cruciata Clade; **AS**, Asperula-Sherardia Clade; **C**, Cymogalia Clade; **G**, Galiinae Clade; **Ga**, Galium Clade; **K**, Kelloggiinae Clade; **R**, Rubiinae Clade.

**Fig 5:**
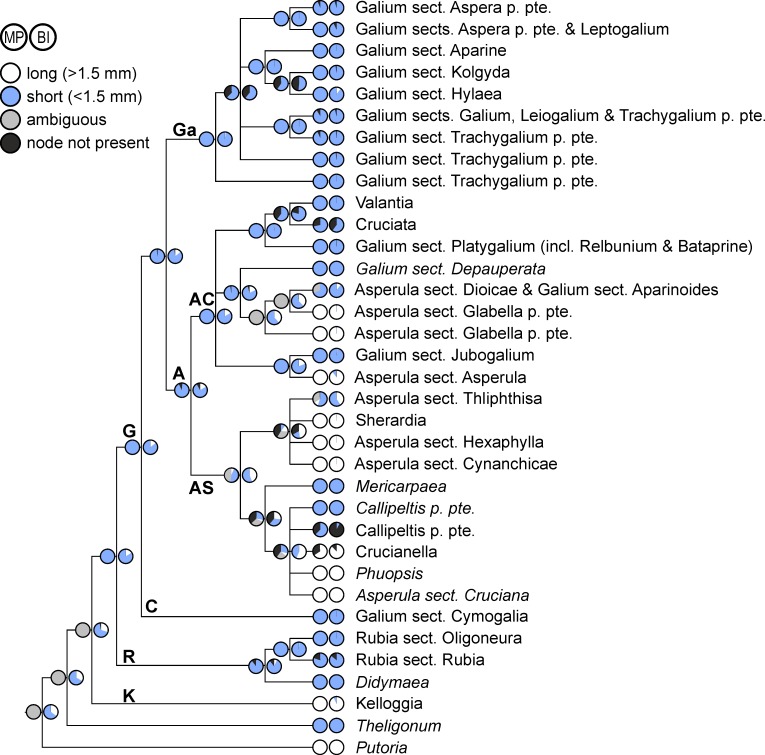
Evolution of corolla tube in Rubieae. Ancestral character states reconstructed over a set of posterior trees using maximum parsimony (left pie charts) and Bayesian inference (right pie charts) are shown on a simplified majority rule consensus tree; therefore, terminal pie charts often show reconstructed character states at the crown node of small terminal clades (indicated by names in normal print) and only rarely the character states of a single accession (indicated by names in italics). Abbreviations of major clades: **A**, Asperula Clade; **AC**, Asperula-Cruciata Clade; **AS**, Asperula-Sherardia Clade; **C**, Cymogalia Clade; **G**, Galiinae Clade; **Ga**, Galium Clade; **K**, Kelloggiinae Clade; **R**, Rubiinae Clade.

**Fig 6:**
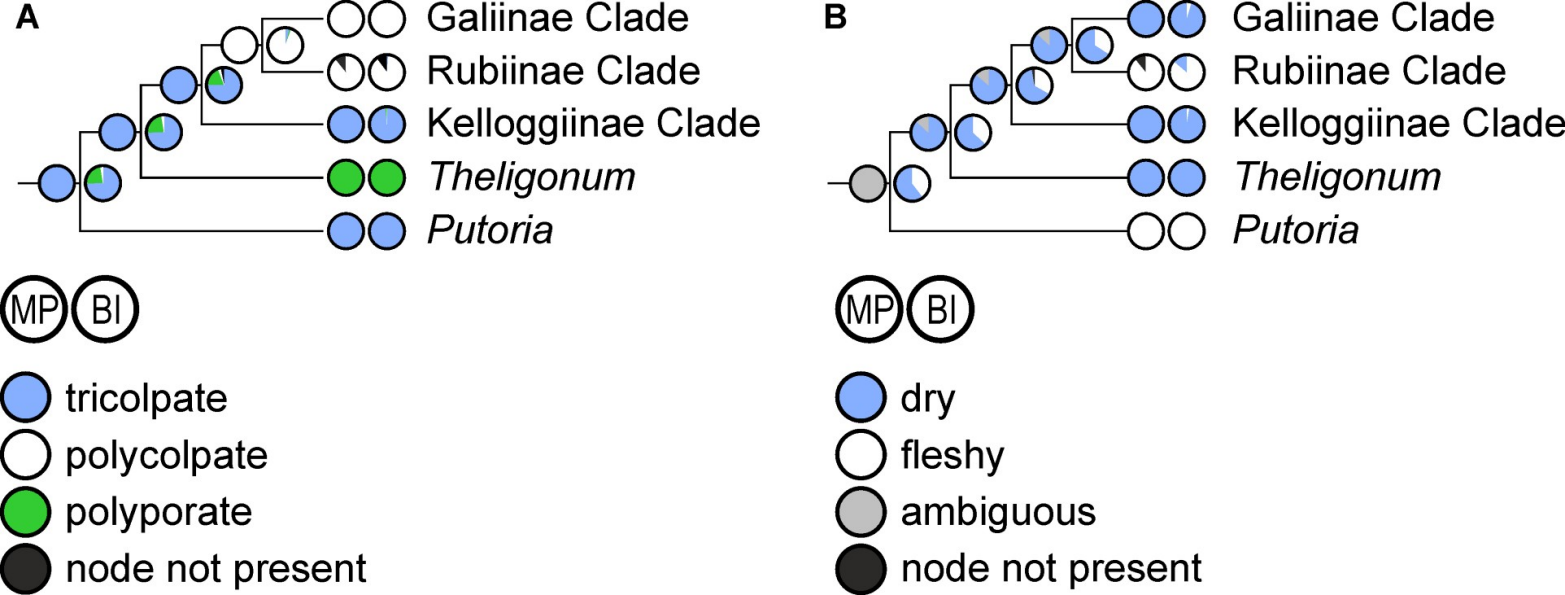
Evolution of (A) pollen type and (B) fruit type in Rubieae. Ancestral character states reconstructed over a set of posterior trees using maximum parsimony (left pie charts) and Bayesian inference (right pie charts) are shown on strongly simplified majority rule consensus trees; note that, therefore, terminal pie charts often show reconstructed character states at the crown node of terminal clades (indicated by names in normal font) and only rarely the character states of a single accession (indicated by names in italics).

**Fig 7:**
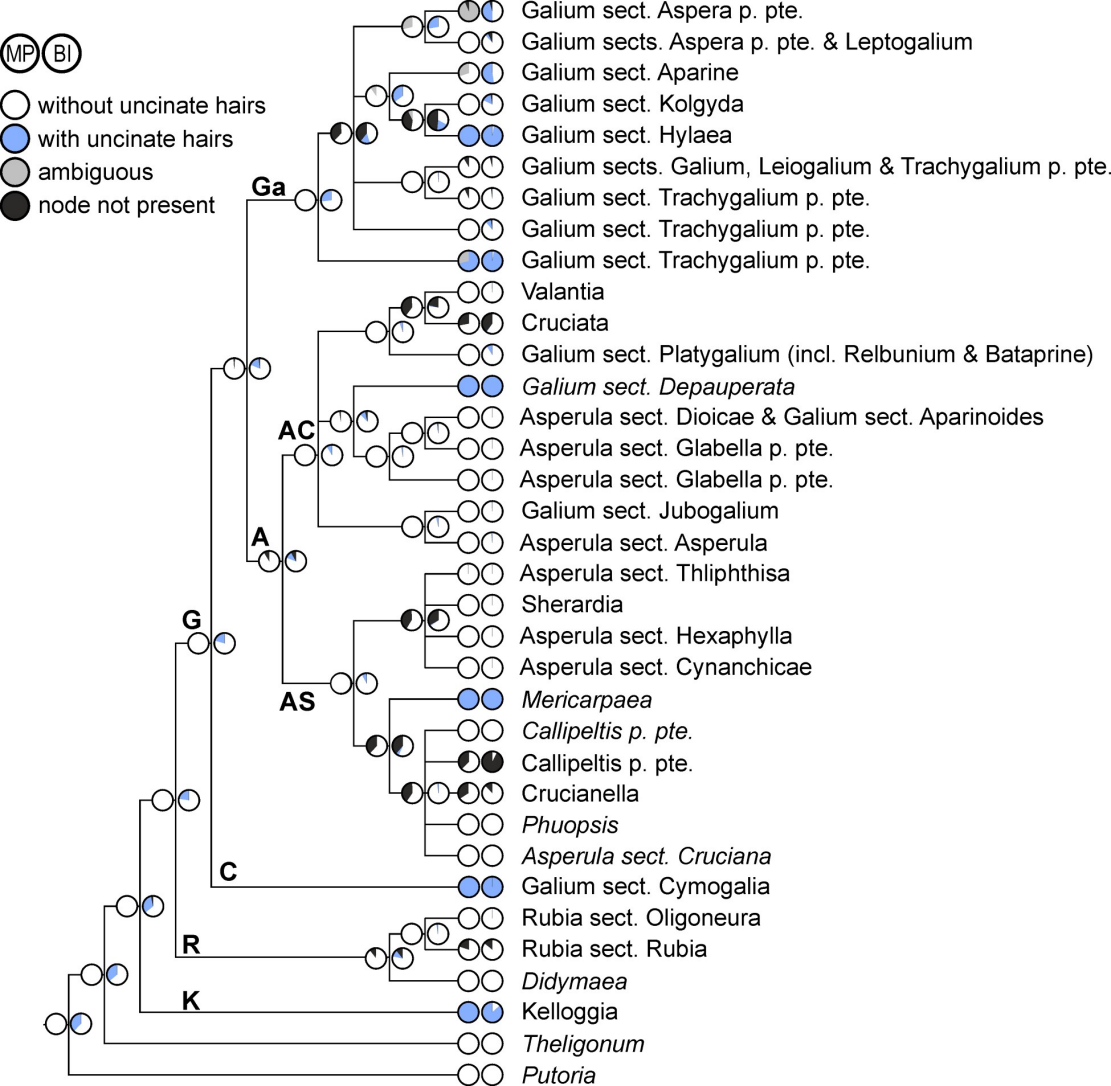
Evolution of fruit indumentum in Rubieae. Ancestral character states reconstructed over a set of posterior trees using maximum parsimony (left pie charts) and Bayesian inference (right pie charts) are shown on a simplified majority rule consensus tree; therefore, terminal pie charts often show reconstructed character states at the crown node of small terminal clades (indicated by names in normal print) and only rarely the character states of a single accession (indicated by names in italics). Abbreviations of major clades: **A**, Asperula Clade; **AC**, Asperula-Cruciata Clade; **AS**, Asperula-Sherardia Clade; **C**, Cymogalia Clade; **G**, Galiinae Clade; **Ga**, Galium Clade; **K**, Kelloggiinae Clade; **R**, Rubiinae Clade.

Character state reconstructions for Rubieae using maximum parsimony and Bayesian inference yield largely congruent results; major discrepancies, if present, are mainly restricted to basal nodes (Figs 3–7). The only exception is life-form: Whereas maximum parsimony reconstructions suggest a perennial Rubieae ancestor and, within the Galiinae Clade, multiple shifts to annuality, an annual ancestor and numerous shifts to perenniality (once each in the Kelloggiinae Clade and the Rubiinae Clade, multiple shifts in the Galiinae Clade) were inferred by Bayesian reconstructions (Fig 3).

Decussate leaves with two small interpetiolar stipules are inferred as ancestral state for the Rubieae as a whole as well as for the Kelloggiinae Clade (Fig 4). Corresponding reconstructions for the Rubiinae Clade, the Galiinae Clade and their common ancestor are burdened with uncertainty (mostly ambiguous reconstructions from maximum parsimony; only moderate nodewise indices of reconstruction precision of 0.499, 0.660 and 0.521, respectively, from Bayesian reconstruction). In both the Rubiinae and the Galiinae Clade, independent shifts from two small stipules to more than two leaf-like stipular elements (i.e., leaf whorls with at least six elements, coded in blue in Fig 4) as well as reversals (at least once in the Rubiinae Clade, multiple times in the Galiinae Clade) to leaf whorls with four equal-sized elements (coded in green in Fig 4) are reconstructed.

Applying a model of ordered character states has strong impacts on the results (Fig 4). These include (1) increasing uncertainty, especially from Bayesian reconstruction, at basal nodes (for example, indices of reconstruction precision of the root node and the node pertaining to Rubieae plus *Theligonum* decrease from 0.923 and 0.927 to 0.351 and 0.347, respectively); (2) higher reconstruction proportions, mostly at basal nodes, of decussate leaves with two nearly leaf-like stipules (coded in orange in Fig 4; reconstructed by maximum parsimony for the Galiinae Clade and having the highest reconstruction probability for basal nodes from Bayesian reconstruction); (3) higher reconstruction proportions, throughout the tree, of leaf-whorls with four equal elements (coded in green in Fig 4; e.g., highest reconstruction probability as ancestral state for *Rubia* and for the Galiinae Clade).

Long corolla tubes, as found in the outgroup *Putoria*, are reconstructed as ancestral only for the Kelloggiinae Clade, whereas short corolla tubes are reconstructed as ancestral for the Rubiinae Clade, the Galiinae Clade and their common ancestor (Fig 5). Within the Galiinae Clade, long corolla tubes are inferred to have evolved multiple times independently. Reconstructions are burdened with high uncertainty towards the root of the tree (ambiguous reconstructions from maximum parsimony and low reconstruction precision of 0.289–0.380 from Bayesian inference).

Polycolpate pollen (Fig 6A) is reconstructed, essentially without ambiguity, as ancestral for the Rubiinae Clade, the Galiinae Clade and their common ancestor. Towards the root of the tree reconstruction precision from Bayesian inference decreases (0.610–0.626), but tricolpate pollen, as in the Kelloggiinae Clade, consistently has the highest probability and is unambiguously reconstructed as ancestral for Rubieae by maximum parsimony.

Dry fruits are reconstructed as ancestral for all clades except the Rubiinae Clade (Fig 6B), but reconstructions become increasingly uncertain towards the root of the tree (ambiguous reconstruction of the root state from maximum parsimony, and reconstruction precision values ranging from 0.213 to 0.340 from Bayesian reconstruction). Within the Galiinae Clade, transitions from dry to fleshy fruits are inferred at a few terminal branches in the Asperula Cruciata Clade, specifically within *Galium* sect. *Platygalium* (S4 Appendix). Uncinate hairs on the fruit (Fig 7) are inferred to have appeared multiple times independently (once in the Kellogginae Clade, several times in the Galiinae Clade) from ancestors with fruits (glabrous or not) without uncinate hairs. Although maximum parsimony and Bayesian reconstruction yield congruent results, the latter is burdened with low reconstruction precision especially at basal nodes (e.g., reconstruction precision of 0.328 for the Rubieae as a whole).

### Biogeography

Age estimates obtained from the analysis of molecular data alone were older than those obtained from the analysis of combined molecular, morphological and biogeographical data (see annotated maximum clade credibility trees available as S3 and S4 Appendices). Age estimates, especially at deeper nodes, were burdened with high uncertainty, causing mean values of one analysis being within the highest posterior density interval of the other analysis. Specific age estimates of a few selected nodes are presented in the Discussion (under “Spatiotemporal Diversification of Rubiaceae”).

**Fig 8:**
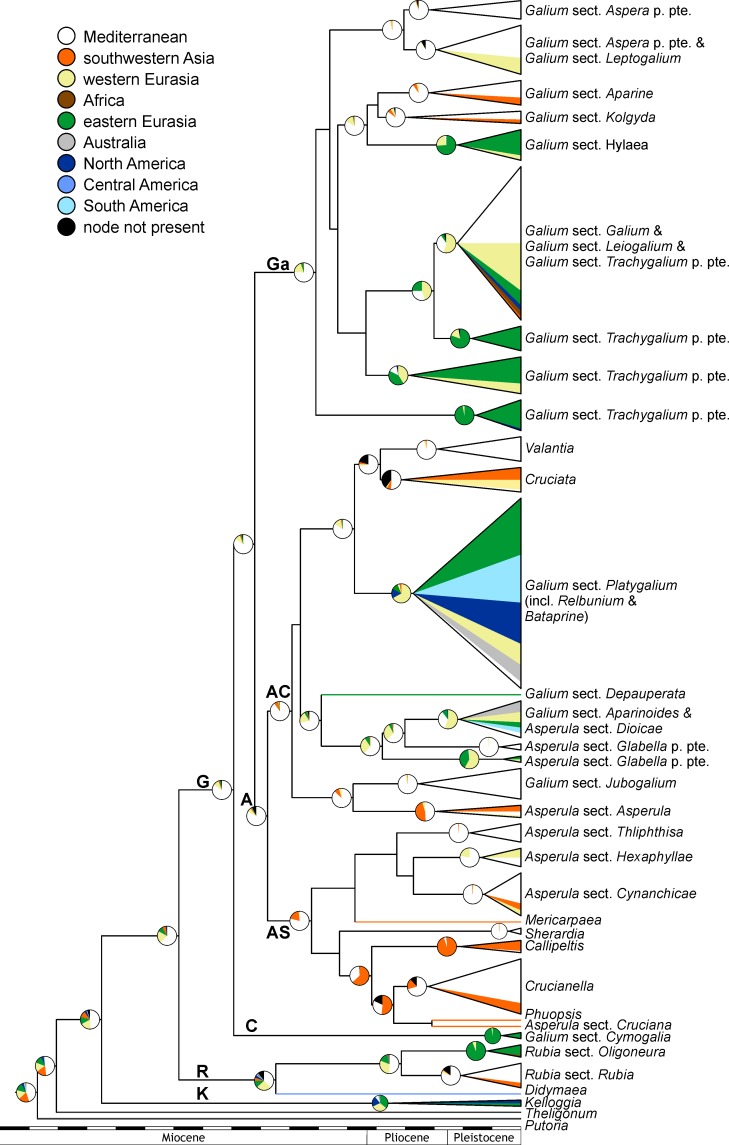
Spatiotemporal evolution of Rubieae. Ancestral areas reconstructed via Bayesian analysis of combined data (sequence data, trait data, biogeographical data) shown on a simplified maximum clade credibility tree (node heights are median ages); reconstructions are shown for all collapsed clades and for nodes that have posterior probability of at least 0.7. Collapsed clades (the same as used in Figs 3–7) are shown as triangles, whose vertical extension is proportional to sample size; the color of the triangles (colors corresponding to those used in the ancestral areas pie charts) indicates the proportion of geographic areas in the distribution of the included taxa. Scale bar is in million years, the duration of geological epochs is indicated. Abbreviations of major clades: **A**, Asperula Clade; **AC**, Asperula-Cruciata Clade; **AS**, Asperula-Sherardia Clade; **C**, Cymogalia Clade; **G**, Galiinae Clade; **Ga**, Galium Clade; **K**, Kelloggiinae Clade; **R**, Rubiinae Clade.

Among the delimited geographic regions, the Mediterranean receives the highest probability for being part of the ancestral range of the Galiinae Clade and the Rubiinae Clade as well as their ancestor; for the Kelloggiinae Clade, eastern Eurasia receives the highest probability (Fig 8). These reconstructions are burdened with some uncertainty (reconstruction precision of 0.351 for the Rubiinae Clade, of 0.716 for the Galiinae Clade, and of 0.271 for the Kelloggiinae Clade). Consequently, ancestral areas with cumulative posterior probabilities of at least 0.8 (i.e., the set of geographic regions, whose posterior probabilities in decreasing order sum up to at least 0.8) include three regions (only two, Mediterranean and western Eurasia, for the Galiinae Clade and for the clade pertaining to Rubiinae Clade plus Galiinae Clade); of those, only western Eurasia is found in the cumulative set for each major clade, including the Kelloggiinae Clade. Both southwestern Asia, eastern Eurasia and the Americas are inferred to have been reached several times independently from European (western Eurasian) and/or Mediterranean ancestors (e.g., in the Galiinae Clade or the Asperula-Cruciata Clade; Fig 8). Multiple colonizations (probably from Europe or eastern Asia) are also inferred for Africa (in *Rubia* sect. *Oligoneura* and *Galium* sect. *Galium* of the Rubiinae and the Galiinae Clade, respectively) and Australia (In *Asperula* sect. *Diocae* and *Galium* sect. *Platygalium*, both in the Asperula-Cruciata Clade), although only few representatives from these areas have been included in this study.

## Discussion

### Current taxonomy does not reflect phylogenetic relationships

It appears to be a common trend in angiosperms, that large traditionally circumscribed genera are identified as non-monophyletic by molecular data (e.g., *Campanula*/Campanulaceae, *Salvia*/Lamiaceae, *Senecio*/Asteraceae; [36–39]). This is also the case in Rubieae, where the large genera *Galium* and *Asperula* are inferred as paraphyletic and polyphyletic, respectively (Fig 1). This goes back to Linnaeus, who used a homoplasious character for their separation: long corolla tube in *Asperula* versus rotate corollas in *Galium* (Fig 5). In contrast, the monophyly of smaller genera is supported (or at least not significantly rejected) by our data. Most of these (*Callipeltis*, *Crucianella*, *Cruciata*, *Mericarpaea*, *Phuopsis*, *Sherardia*, *Valantia*) are phylogenetically nested within *Galium* or *Asperula* as part of the Asperula Clade (clade **A** in Fig 1).

Ignoring the possibility of maintaining paraphyletic genera as occasionally advocated [40], there are two alternatives to taxonomically resolve the issues around the Linnean genera *Galium* and *Asperula*. The first, taxonomic lumping, is to recognize a broadly defined *Galium* thus including the entire Galiinae Clade (clade **G** in Fig 1) with all its current genera. This would render *Galium* morphologically very heterogeneous without eliminating the need for taxonomic recognition of smaller clades, albeit now at the infrageneric level. The second possibility, taxonomic splitting, is to use more narrowly circumscribed genera, thus retaining all of the currently recognized smaller genera and adding new genera for several well supported clades of *Galium* and *Asperula*. This alternative is facilitated by the reasonably good correspondence of currently recognized, morphologically defined taxonomic sections of *Galium* and *Asperula* with herein identified clades. Specifically, seven out of twelve studied sections of *Galium* (e.g., *G*. sect. *Jubogalium*, *G*. sect. *Platygalium*) and five out of seven studied sections of *Asperula* (e.g., *A*. sect. *Asperula*, *A*. sect. *Cynanchicae*) are supported by molecular data (Figs 1 and 2). As both possibilities, lumping and splitting, are congruent with phylogenetic relationships, the eventual choice should be rather based on pragmatic considerations (e.g., diagnosability, amount of nomenclatural changes). Taxonomic lumping would result in a huge genus *Galium* and the elimination of several well established and readily recognizable genera (e.g., *Crucianella*, *Sherardia*, *Valantia*). Therefore, we regard future moderate splitting and the establishment of about ten well supported new genera as preferable.

Lack of monophyly of the traditional genera *Galium* and *Asperula* and the phylogenetic structure within the Galiinae Clade (Fig 1) fully agrees with relationships inferred by Soza and Olmstead [10] and by Yang et al. [13] (Table 1). Their analyses did not include a few important taxa (*Mericarpaea*, *G*. sect. *Jubogalium*, *Asperula* sect. *Dioicae*; in case of [10] also not *G*. *paradoxum*), but were based on more markers, resulting in overall higher resolution. Thus, the dichotomy within the Asperula-Sherardia Clade into the clade containing *Phuopsis* and *Callipeltis* (clade IV-E of Soza and Olmstead [10]) sister to the clade containing several *Asperula* sections and *Sherardia* (clade IV-F of Soza and Olmstead [10]) is strongly supported [10,13]. Additionally, the relationships among the three clades within the *Asperula-Cruciata* Clade are fully resolved. Specifically, the clade including *Asperula* sect. *Glabella* and *Galium* sect. *Depauperata* (clade V in Soza and Olmstead [10], clade VIII in Ehrendorfer and Barfuss [12], clade III-b in Yang et al. [13]: Table 1) is sister to the clade comprising *Asperula* sect. *Asperula* plus *Galium* sect. *Jubogalium* (and probably also *Microphysa* [13] not included by us; clade VI in Soza and Olmstead [10], clade VII in Ehrendorfer and Barfuss [12], clade III-c in Yang et al. [13]: Table 1) and the clade including *Valantia*, *Cruciata* plus *Galium* sect. *Platygalium* (including the former genera *Relbunium* and *Bataprine*; clade VII of Soza and Olmstead [10], clade IX in Ehrendorfer and Barfuss [12], clades III-d and III-e in Yang et al. [13]: Table 1). Soza and Olmstead [10] did not include *Galium paradoxum* of the monotypic series *Paradoxa* within *Galium* sect. *Cymogalia* [41,42]. This species is inferred as sister to all other taxa of the Galiinae Clade (Fig 1) in congruence with previous results [13,43], and was only recently separated as a new genus *Pseudogalium* [13]. Given that this species has two opposite leaves and two distinctly smaller, yet leaf-like interpetiolar stipules, its inclusion is important for a better understanding of the evolution of multi-parted leaf whorls in the whole tribe (see section “Character evolution”).

Sister group to the Galiinae Clade is the Rubiinae Clade (taxonomically already recognized as subtribe Rubiinae s. str.: [9]), comprising the Old World *Rubia* and the New World *Didymaea* (clades II and I, respectively, in Soza and Olmstead [10]. Table 1). The two genera share fleshy mericarps (that differ from those observed in some species of *Galium* sect. *Platygalium* within the Asperula-Cruciata Clade; Fig 6B, see section “Character evolution”), lack of hooked hairs (Fig 7) and pluricolpate pollen (Fig 6A; [9,44]).

Sister group to the rest of Rubieae is *Kelloggia*, sole member of the Kelloggiinae Clade (Fig 1), taxonomically already recognized as subtribe Kelloggiinae [9]. *Kelloggia* contains only two herbaceous perennial species, which exhibit an intercontinental disjunction between the high mountains of SW-China and montane forests of western North-America. The inclusion of *Kelloggia* in Rubieae is supported by the presence of stiff and hooked hairs on the mericarps, a feature common in some, but not all Rubieae clades (Fig 7) that is essentially absent in other tribes of the family except three species of *Pterogaillonia* and *Pseudogaillonia* from SW-Asia [45] now accommodated in *Plocama* within the Putorieae, sister tribe of Rubieae [22]. *Kelloggia* has a persistent and non-reduced calyx and tricolpate pollen grains (the likely ancestral condition in Rubieae; Fig 6A), while other species of Rubieae have usually reduced calyces and always polycolpate pollens [9,46,47].

Sister group to all Rubieae is the genus *Theligonum* (Fig 1), formerly classified as an undisputed separate family Theligonaceae. Based on molecular evidence, Robbrecht and Manen [9] merged tribe Theligoneae as subtribe Theligoninae into an extended tribe Rubieae. While this is fully consistent with phylogenetic relationships and avoids the recognition of a species-poor tribe (only a single genus with four species: [48]), we favour recognition of this morphologically strongly aberrant clade as a separate tribe, because no morphological synapomorphies support such an extension of Rubieae.

### Character evolution

#### Life form

Life form, i.e., perennial versus annual, has changed multiple times within the Galiinae Clade, whereas both the Rubiinae and the Kelloggiinae Clades have exclusively perennial members (Fig 3). This indicates a high plasticity of this character, permitting lineages to evolve into habitat types with unpredictable conditions, such as dry or ephemeral habitats, where annuality may be advantageous [49]. The ancestral life history in Rubieae remains, however, somewhat ambiguous. Whereas maximum parsimony reconstructs perenniality as ancestral, Bayesian reconstruction infers annuality as ancestral (Fig 3). Although evolution of perennial species from short-lived ancestors has rarely been found in other angiosperms (e.g., *Androsace*/Primulaceae: [50]), this result from a model-based reconstruction for Rubieae may be biased, as we could only include the Mediterranean annual representative of *Theligonum* (the sister lineage to Rubieae), but none of its East Asian perennial members. As members of the consecutive sister groups to Rubieae (i.e., *Putorieae*, *Saprosma*, and *Paederieae*) are all perennial [22], the result from the maximum parsimony reconstruction of a perennial ancestor of *Rubieae* appears more plausible.

#### Leaf whorls

A prominent feature of many species in Rubieae are whorls of four to more than ten leaf-like elements. Two of those correspond to the true opposite leaves that can bear axillary shoots, but the identity of the remaining elements is somewhat disputed (reviewed in [14]). The presence of interpetiolar stipules in other Rubiaceae, however, suggests that those leaf-like elements are evolutionarily derived from stipules, even though they may show features of true leaves (e.g., separate vascular traces in *Galium rubioides*: [14]) and thus could be considered to be intermediate between leaves and stipules. Irrespective of this ambiguity, the lack of more than two true (i.e., potentially shoot-bearing) leaves per whorl in Rubieae (rarely three true leaves in the multi-parted upper whorls of *Phuopsis stylosa* and regularly three true leaves in the three-leaved whorls of *Rubia fruticosa* [51], the latter not included in our study) allows leaf-like elements beyond the two true leaves to be treated as homologous to stipular elements.

Early authors (e.g., [52]) favored the hypothesis that whorls with six and more elements originate from four-leaved whorls, but a recent study inferred whorls with at least six components as ancestral [10]. Here we show that the inferred sequence of leaf whorl development is strongly affected by the statistical reconstruction method (parsimony versus likelihood) and particularly by the transitions allowed between character states (unordered versus ordered character state changes). Specifically, under a model of ordered character state changes, four-parted whorls (i.e., two leaves and two at least strongly enlarged or even fully leaf-like interpetiolar stipules, coded in orange and green, respectively, in Fig 4) are suggested as ancestral for the Galiinae Clade (Fig 4). In contrast, under a model of unordered character state changes, whorls with at least six elements are inferred as ancestral for the Galiinae Clade (high ambiguity in parsimony reconstructions; Fig 4). Irrespective of the model used, multi-parted whorls in Rubieae probably were derived from a setting of two opposite leaves with two small interpetiolar stipules, a character state found, for instance, in the Kelloggiinae Clade or in *Theligonum* (Fig 4). Thus, none of our reconstructions corroborate the finding of Soza and Olmstead [10] that multi-parted leaf whorls are ancestral for the entire Rubieae. This may be due to different codings: Whereas we as well as Yang et al. [13] coded *Didymaea* and *Kelloggia* as possessing small interpetiolar stipules (that may be bifid: [30,53]), Soza and Olmstead [10] coded them, seemingly erroneously, as possessing at least six elements per whorl. They also coded their outgroup taxa *Staelia*, *Spermacoce* and *Galianthe* (not included here) as possessing six or more leaf whorl parts, thus further reinforcing reconstruction of an ancestrally multi-parted leaf-whorl in their analyses. However, the leaf structure of these genera is usually interpreted as a pair of opposite leaves with two more or less sheath-like fimbriate interpetiolar stipules, characteristic for the entire tribe Spermacoceae in its narrow circumscription [1,54]. In summary, we do not find support for the hypothesis of a primary origin of multi-parted leaf whorls, but rather an origin from opposite leaves with two interpetiolar stipules that are subsequently enlarged and increased in their number, in line with recent inferences [13]. This sequence of evolution is supported by two lines of evidence: (1) In subfamily Rubioideae, where the tribe Rubieae belongs to, stipules always originate as single (but sometimes ± divided) interpetiolar stipules from a meristem that links the opposite leaf bases on both sides of the axis [1,14,52]. Thus, whorls with four elements can be readily interpreted as opposite leaves with two enlarged interpetiolar stipules (e.g., in *Galium paradoxum*). (2) During the ontogeny of shoot development Rubieae taxa possessing whorls of six or more elements always pass through an early stage of leaf whorls with only four elements [52,55].

Irrespective of the above discussed ancestral state in Rubieae as a whole, statistical analyses suggest that within the Galiineae Clade four-parted whorls have evolved from whorls with at least six elements independently in *Callipeltis*, *Asperula* sect. *Cynanchicae*, *A*. sect. *Glabella* and the clade comprising *Cruciata*, *Valantia* and *Galium* sect. *Platygalium* (Fig 4). This is somewhat counterintuitive as one would expect whorls with a stabilized number of elements, as is the case for the four-parted whorls of, for instance, *Galium* sect. *Platygalium*, to be more plesiomorphic than whorls with unstable numbers of elements, as is the case for the whorls of, for instance, *Galium* sect. *Galium* with six or more elements. This notwithstanding, four-parted whorls with stipules that are smaller than the leaf blades as observed in *Galium paradoxum* and *G*. sect. *Depauperata* appear to be homoplasious, being an ancestral state in the former, but a derived state in the latter (Fig 4). Secondary reduction may explain the presence of species in *Crucianella*, *A*. sect. *Cruciana* (such as *A*. *anatolica* not included in our study), and *G*. sect. *Aparinoides* with four-parted whorls (such as *G*. *saturejifolium* not included in our study) as well as the presence of species with opposite leaves and small to nearly absent stipules in some species of *Asperula* sect. *Dioicae* (*A*. *gemella* and *A*. *geminifolia*: [56]; these species are not included in our study) and in the Southwest Asian species of *Asperula* sect. *Oppositifoliae* (not included in our study, but most likely closely related to *A*. sect. *Cynanchicae*: [45]). An extended sampling within these groups will, however, be necessary to ascertain the direction of leaf evolution (reduction versus increase of elements).

#### Flower and fruit characters

The presence of a long corolla tube has been used as taxonomic character to distinguish *Asperula* from *Galium* (e.g., [45]). This character is highly homoplasious within Rubieae (Fig 5). Due to differences in the relative contribution of developmental processes involved in corolla tube formation (i.e., formation of a stamen-corolla tube, formation of a corolla tube sensu stricto, and postgenital fusion of petals), similar looking tubular corollas may actually constitute different character states [57]. Although the widespread occurrence of long corolla tubes in Rubiaceae outside Rubieae suggests that a long corolla tube is the ancestral state, this may not be true for Rubieae. The sister group of Rubieae, the morphologically strongly aberrant *Theligonum*, has short corolla tubes, rendering reconstructions at the crown node of Rubieae uncertain, especially in maximum parsimony reconstructions. Notwithstanding this uncertainty, statistical analyses infer that within Rubieae long corolla tubes may have evolved from short corolla tubes multiple times, especially in the Galiinae Clade (Fig 5). Shifts in corolla tube length may be associated with different pollinators, but relevant data from Rubieae are still rather scarce. The rotate flowers of *Galium* species are unspecialized and visited by lepidopterans, beetles, flies, ants, wasps and bees [58], whereas species with longer corolla tubes may have a more specific set of pollinators.

Within Rubieae fruit type (Fig 6B) changed from dry to fleshy fruits once in the Rubiinae Clade and at least four times independently [11] within the New World members of the Asperula-Cruciata Clade (e.g., the former genera *Relbunium* [59] and *Bataprine* [60], both nested within *Galium* sect. *Platygalium*). Homology of fleshy fruits is, however, uncertain as fleshy fruits may differ anatomically (e.g., multi-layered versus few-layered fleshy mesocarps in *Rubia* versus *Relbunium*: [59]) or fruits may change their appearance during development (e.g., juvenile fleshy fruits versus mature dry mericarps in *Putoria*: [22]).

Fruit surface structure (i.e., the absence or presence of uncinate hairs) changed multiple times and is highly homoplasious (Fig 7). Specifically, uncinate hairs appear independently in the Kelloggiinae Clade and the Galiinae Clade, among the latter in the Cymogalia Clade and particularly the *Galium* Clade, less often the *Asperula* Clade (Fig 7). The genetic basis for formation of uncinate hairs is not known, but may have evolved only once in the early Rubieae (i.e., after the divergence of *Theligonum* from the rest) with subsequent switching on or off in different lineages. Uncinate hairs allow diaspores to be dispersed epizoochorically, although possibly only in combination with other traits such as small diaspore mass [61]. Epizoochory is considered a major mechanism of long-distance dispersal [62,63], and may have contributed to the worldwide distribution of *Galium* weeds, such as *Galium aparine* and *G*. *spurium*. Straight fruit hairs occur in species of *G*. sects. *Platygalium* and *Jubogalium*, where they may contribute to anemochorous dispersal.

### Spatiotemporal diversification of Rubieae

The geographic origin of Rubieae is uncertain. The Mediterranean has the highest probability (Fig 8), but there might be a bias due to the outgroups *Putoria* (now part of *Plocama*) and *Theligonum*, from which only Mediterranean representatives were included. But even without these outgroups, high geographic complexity in the Kelloggiinae Clade and the Rubiinae Clade renders identification of the ancestral area for the entire Rubieae difficult. The Kelloggiinae Clade exhibits an East Asian–western North American disjunction. The Rubiinae Clade is essentially found on all continents except Australia with an early Old World–New World vicariance in *Rubia–Didymaea* and a western Eurasian (Mediterranean, southwestern Asia)–mainly eastern Eurasian (eastern Asia) vicariance in *Rubia* (Fig 8).

The timing of these splits is also uncertain. Although the prior on the stem node age of Rubieae was set to have its mean at 29 Ma, the posterior age was much younger (mean age without / with trait data of 21.9 / 16.4 Ma). This is likely due to the second calibration (the split of the two *Kelloggia* species), as our estimates agree quite well with those of Nie et al. [30]. Specifically, the stem node age of Rubieae was (given as mean and, in parentheses, the highest posterior density interval from analyses without / with trait data) 21.9 (12.3–33) / 16.4 (10–23.6) Ma versus around 23 Ma in Nie at al. [30]; the crown node age of Rubieae was 19.6 (10.8–29.8) / 14.9 (9.2–21.7) Ma versus about 17 Ma in Nie at al. [30], and the crown node age of the clade comprising the Rubiinae Clade plus the Galiinae Clade was 16.4 (9–25) / 12.2 (7.4–17.7) Ma versus about 13 Ma in Nie at al. [30]. These estimates are only slightly younger than those obtained by Wikström et al. ([64]; stem and crown node ages of Rubieae of 23–28 (13–38) Ma and 18–23 (9–33) Ma, respectively), but much younger than those inferred by Deng et al. ([65]; a crown node age of Rubieae of 35.57 (29.27–42.99) Ma), which is likely due to the use of different sets of fossils for calibration (17 fossil calibrations exclusively outside Rubiaceae by Wikström et al. [64] versus four fossil calibrations exclusively within Rubiaceae by Deng et al. [65]).

Ages inferred by Wikström et al. [64] and by us place the divergence times of the Kelloggiinae Clade, the Rubiinae Clade and the Galiinae Clade in the Miocene (possibly extending into the Oligocene). Although a continuous Atlantic land bridge between Europe and North America did not exist anymore in the Eocene, there is evidence from both biogeographic and paleobotanical studies that discontinuous “land bridges” may have existed up to the late Miocene [66,67]. A thus envisaged stepping stone dispersal across the North Atlantic may also be invoked for explaining the disjunction between *Rubia* and *Didymaea*, whose split is estimated to have occurred around 11.9 (4.8–19.9) / 8.8 (4–14) Ma, although the presence of fleshy fruits renders long-distance dispersal via birds [68] a plausible alternative.

Diversification in the Galiinae Clade also started in the Miocene around 14 (7.7–21.4) / 10.2 (6.2–14.9) Ma probably in the Mediterranean (Fig 8). From there, eastern Eurasia was reached multiple times. This started probably already in the Miocene (the sister species to all others in this clade, *Galium paradoxum*, is restricted to eastern and central Eurasia), but happened mainly in the Pliocene and Pleistocene in the Galium Clade and the Asperula-Cruciata Clade (Fig 8); for instance the stem node age for the clade including species of *Galium* sect. *Platygalium* is estimated to be 8.3 (4.1–13.1) / 5.9 (3.3–8.8) Ma. Migration into the New World during these time periods is reconstructed to have occurred from Europe (Fig 8), possibly via eastern Eurasia. Although colonization via a Beringian land bridge cannot be entirely excluded, colonization of America from Europe and/or western Africa via long-distance dispersal was suggested for *Anemone* sect. *Anemone* (Ranunculaceae) in the late Miocene [69] and for *Hypochaeris* (Asteraceae) in the Pliocene to Pleistocene [70]. Colonization of Australia is generally more recent (e.g., the stem node age for Australian *Galium ciliare*, *G*. *gaudichaudii*, and *G*. *migrans* is uncertain, but maximally 3.2 (1.4–5.4) / 2.4 (1.1–3.8) Ma) dating back to the (late) Pliocene to Pleistocene and probably occurred via southeastern Asia.

In contrast to the Galium Clade and the Asperula-Cruciata Clade, which have a strong “mesic” component, i.e., their members are often distributed in temperate to boreal regions of the Old and New World, the Asperula-Sherardia Clade has a more “xeric” aspect with its members being found in the Mediterranean and adjacent southwestern Asia (Fig 8). Its diversification dates back to the (late) Miocene (10.4 (5.5–16.1) / 7.5 (4.5–11) Ma). This diversification may have been triggered by increasing aridification and seasonality. Although this became particularly pronounced only in the middle Pliocene, a proto-Mediterranean-type climate probably already existed in the middle to late Miocene [71].

## Conclusions

Results from analyses of molecular data ([5–7,10–13], this study) converge on a stable picture of phylogenetic relationships within the Rubieae. Specifically, (i) the tribe includes three major clades, the Kelloggiinae Clade (*Kelloggia*), the Rubiinae Clade (*Didymaea*, *Rubia*) and the most species-rich Galiinae Clade (*Asperula*, *Callipeltis*, *Crucianella*, *Cruciata*, *Galium*, *Mericarpaea*, *Microphysa*, *Phuopsis*, *Sherardia*, *Valantia*); and (ii) within the Galiinae Clade, the largest genera *Galium* and *Asperula* are para- and polyphyletic, respectively, whereas smaller clades correspond to currently recognized taxa (small genera or sections within genera). Eventually, the improved understanding of phylogenetic relationships should be translated into taxonomy, as was recently done for *Galium paradoxum* moved to the novel genus *Pseudogalium* [13].

Several characters that have been traditionally used for taxonomic purposes (e.g., life-form, flower shape, fruit characters) are highly homoplasious and have changed multiple times independently. This agrees with the hypothesis that these characters may be involved in adaptation to deviating ecological conditions, such as annuality in climates with longer dry periods, differences in corolla tube length in response to different pollinator guilds, and fruits that are fleshy or possess uncinated hairs as means for endozoochory or ectozoochory, respectively. More data will, however, be necessary to establish causal relationships between trait shifts and ecological shifts in Rubieae. Whereas the hypothesis on the origin of leaf whorls, a characteristic feature of the entire tribe, from opposite leaves with two small interpetiolar stipules that are subsequently enlarged and increased in their number [13] has been recently challenged [10], we provide additional support for it.

Applying Bayesian ancestral area reconstruction and molecular dating, early diversification of Rubieae is inferred as having started during the Miocene in western Eurasia, with early disjunctions between the Old and the New World possibly being due to connections via a North Atlantic land bridge. Diversification of the Galiineae Clade, the most species-rich group of Rubieae, started later in the Miocene, probably in the Mediterranean, from where lineages reached, often multiple times, Africa, eastern Asia and further on the Americas and Australia. Given considerable uncertainty in the age estimates and ambiguity in biogeographic reconstructions, a denser sampling within Rubieae ideally employing a broader set of calibration points will be necessary to refine and test these biogeographic hypotheses.

## Supporting information

S1 Fig Phylogenetic relationships of Rubieae inferred from maximum parsimony analysis of the complete data set. Shown is the strict consensus tree with bootstrap values. Abbreviations of major clades: AS, Asperula-Sherardia Clade; C, Cymogalia Clade; G, Galiinae Clade; Ga, Galium Clade; K, Kelloggiinae Clade.
(PDF)

S1 Table Samples analyzed. Investigated taxa, collecting information, GenBank accession numbers, biogeographic distribution and trait values (life-form, leaf whorls, corolla type, pollen type, fruit consistence and fruit indumentum with respect to uncinate hairs).
(PDF)

S1 Appendix BEAST input file (xml-file) of combined data set (molecular, morphological and biogeographic data). (XML)

S2 Appendix Mesquite input file (nexus-file) including DNA sequence and trait data as well as phylogenetic trees (posterior trees and majority-rule tree to display results on). (ZIP)

S3 Appendix Annotated maximum clade credibility tree from analysis of the molecular data only. (TXT)

S4 Appendix Annotated maximum clade credibility tree from analysis of the combined data set (molecular, morphological and biogeographic data). (TXT)
