# Correction: Phylogeny, character evolution and spatiotemporal diversification of the species-rich and world-wide distributed tribe Rubieae (Rubiaceae)

**DOI:** 10.1371/journal.pone.0211589

**Published:** 2019-01-25

**Authors:** Friedrich Ehrendorfer, Michael H. J. Barfuss, Jean-Francois Manen, Gerald M. Schneeweiss

The funding section is incorrect. The correct funding information is as follows: Open access funding provided by University of Vienna.
