# Supplementary figures and images for: Phylogeny, character evolution and spatiotemporal diversification of the species-rich and world-wide distributed tribe Rubieae (Rubiaceae)
Source: PLoS One. 2018 Dec 5;13(12):e0207615. doi: 10.1371/journal.pone.0207615 (PMC6281350; doi:10.1371/journal.pone.0207615)

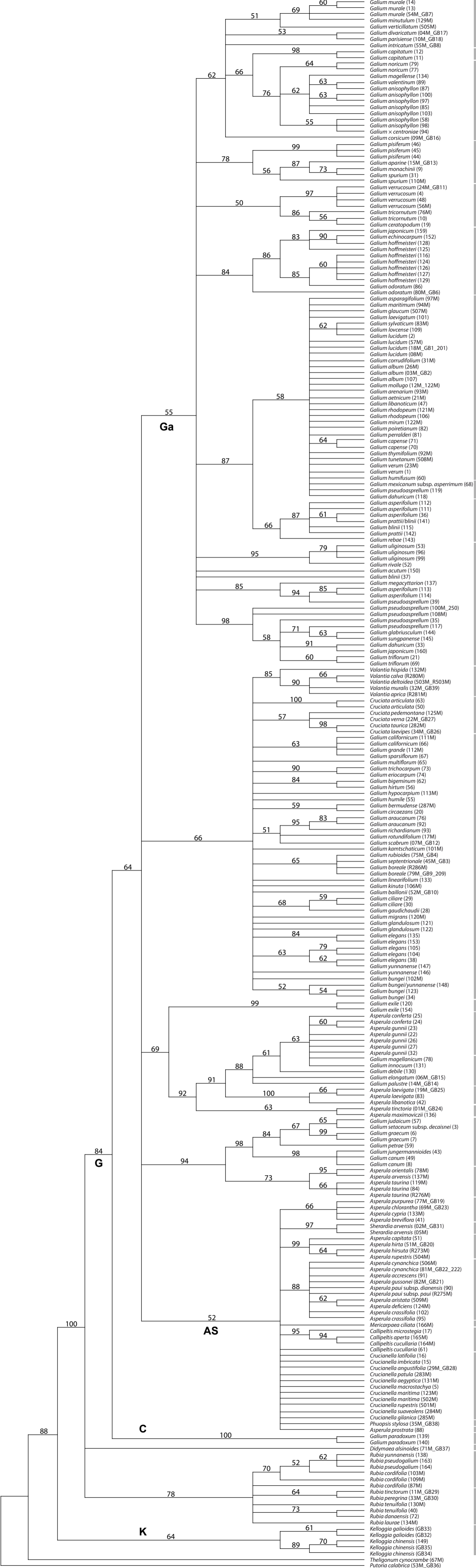

Supplement: S1 Fig — Shown is the strict consensus tree with bootstrap values. Abbreviations of major clades: AS, Asperula-Sherardia Clade; C, Cymogalia Clade; G, Galiinae Clade; Ga, Galium Clade; K, Kelloggiinae Clade. (PDF) [file pone.0207615.s001.pdf]
