# Supplementary material for: Phylogeny, character evolution and spatiotemporal diversification of the species-rich and world-wide distributed tribe Rubieae (Rubiaceae)
Source: PLoS One. 2018 Dec 5;13(12):e0207615. doi: 10.1371/journal.pone.0207615 (PMC6281350; doi:10.1371/journal.pone.0207615)
Supplement: S1 Table — Investigated taxa, collecting information, GenBank accession numbers, biogeographic distribution and trait values (life-form, leaf whorls, corolla type, pollen type, fruit consistence and fruit indumentum with respect to uncinate hairs). (PDF) [file pone.0207615.s002.pdf]

S1 Table. Samples analyzed.

Investigated taxa, collecting information, GenBank accession numbers (235 *atpB-rbcL* sequences, 136 *rpl32-trnL* sequences; N/A = not available), biogeographic distribution and trait values (life-form, leaf whorls, corolla type, pollen type, fruit consistence and fruit indumentum with respect to uncinate hairs).

Taxon (sample number)	Locality (voucher information)[a]	GenBank accession numbers		Distribution	Life form	Elements per leaf whorl	Corolla	Pollen	Fruit consistence	Uncinate hairs on fruits
		atpB-rbcL	rpl32-trnL							
**outgroups**										
Putoria *calabrica* (L. f.) DC. (53M_GB36)	Greece: Ionian Islands (WU, G: FE 930409-2301)	MK028086	N/A	Mediterranean	perennial	2 + 2 small stipules	tubular	3-colpate	fleshy	no
Theligonum *cynocrambe* L. (67M)	Greece: Ionian Islands (WU: FE 930416-6201)	MK028091	N/A	Mediterranean	annual	2 + 2 small stipules	rotate	polyporate	dry	no
Asperula										
**sect. *Asperula***										
A. arvensis L. (137M)	Cyprus: Ayia-Anna, Vyzakia (G: Iter Mediterranea IV 1479, Cyprus)	MK028049	N/A	Mediterranean	annual	6 or more	tubular	polycolpate	dry	no
A. orientalis Boiss. & Hohen. (78M)	Switzerland: Geneva, Bot. Garden (G: A. Natali s.n., Geneva Bot. Garden 780237/0)	MK028097	N/A	SW Asia	annual	6 or more	tubular	polycolpate	dry	no
A. taurina L. (119M)	Central Europe (no herbarium voucher)	MK028034	N/A							
A. taurina L. (84)	Switzerland: Zürich, Bot. Garden (WU: FE, 2.6.2001)	MK027952	MK027815	Europe & SW Asia	perennial	4	tubular	polycolpate	dry	no
A. taurina L. (R276M)	Italy: Prov. Cuneo (G: A. Charpin 23296)	MK028110	N/A							
**sect. *Cruciana***										
A. prostrata (Adams) K.Koch (88)	Georgia: Caucasus, Kasbegi (WU: FE, July 1997)	MK027954	MK027819	SW Asia	perennial	6 or more	tubular	polycolpate	dry	no
**sect. *Cynanchicae***										
A. accrescens Klokov (91)	Georgia: Caucasus, Kasbegi (WU: FE, July 1997)	MK027956	MK027822	SW Asia	perennial	4	tubular	polycolpate	dry	no

A. aristata L. f. (509M)	Italy: Sicilia, Palermo, Monte Rosa (PAL: L. Robba 2, Oct. 2003)	MK028083	N/A	Mediterranean	perennial	4	tubular	polycolpate	dry	no
A. crassifolia L. (102)	Italy: Capri (WU: FE, 10.11.2000 [as "A.tomentosa Ten."])	MK027963	MK027833							
A. crassifolia L. (95)	Italy: Capri (WU: FE, 10.11.2000)	MK027959	MK027826	Mediterranean	perennial	4	tubular	polycolpate	dry	no
A. cynanchica L. (506M)	Italy: Sicilia, Palermo, Madonie (PAL: L. Robba 14)	MK028080	N/A							
A. cynanchica L. (81M_GB22_222)	Switzerland: Geneva, Bot. Garden (G: A. Natali & J.-F. Manen s.n., Geneva Bot. Garden 861771/0)	MK028100	N/A	Europe	perennial	4	tubular	polycolpate	dry	no
A. deficiens Viv. (124M)	Italy: Sardinia, Tavolara (G: Corrias & Diana s.n.)	MK028040	N/A	Mediterranean	perennial	4	tubular	polycolpate	dry	no
A. gussonei Boiss. (82M_GB21)	Switzerland: Geneva, Bot. Garden (G: A. Natali & J.-F. Manen s.n., Geneva Bot. Garden 783214/0)	MK028101	N/A	Mediterranean	perennial	4	tubular	polycolpate	dry	no
A. paui Font Quer subsp. *paui* (R275M)	Spain: Baleares, Ibiza, San Mateo (G, GE: Font Quer 2887)	MK028109	N/A	Mediterranean	perennial	4	tubular	polycolpate	dry	no
A. paui subsp. *dianensis* (Font Quer) De la Torre, Alcaraz & M.B. Crespo (90)	Spain: Alicante, Cabo de San Antalio (WU: M. Boşcaiu)	MK027955	MK027821	Mediterranean	perennial	4	tubular	polycolpate	dry	no
**sect. *Dioicae***										
A. conferta Hook. f. (24)	Australia: NSW, Woodsreef mine (NSW, WU: J.R. Hosking 2773)	MK027904	MK027767	Australia	perennial	6 or more	tubular	polycolpate	dry	no
A. conferta Hook. f. (25)	Australia: NSW, Southern Tablelands (NSW, WU: B.G. Briggs 9766, 9.2.2007)	MK027905	MK027768							
A. gunnii Hook. f. (22)	Australia: NSW, Barrington Tops (NSW, WU: J.R. Hosking 2818)	MK027902	MK027765	Australia	perennial	6 or more	tubular	polycolpate	dry	no

A. gunnii Hook. f. (23)	Australia: NSW, Barrington Tops (NSW, WU: J.R. Hosking 2820)	MK027903	MK027766							
A. gunnii Hook. f. (26)	Australia: NSW, Southern Tablelands (NSW, WU: B.G. Briggs 9759, 8.2.2007)	MK027906	MK027769							
A. gunnii Hook. f. (27)	Australia: NSW, Southern Tablelands (NSW, WU: B.G. Briggs 9744, 4.2.2007)	MK027907	MK027770							
A. gunnii Hook. f. (32)	Australia: Tasmania, Cradle Valley (NSW 744764, WU: B.G. Briggs 9789, 24.2.2007)	MK027912	MK027775							
**sect. *Glabella***										
A. laevigata L. (19M_GB25)	Italy: Elba Island (G: A. Natali & M.-A. Thiébaud s.n.)	MK028057	N/A							
A. laevigata L. (83)	Tunisia: Ain Drahaen (WU: FE, 19.4.2001)	MK027951	MK027814	Mediterranean	perennial	4	tubular	polycolpate	dry	no
A. libanotica Boiss. (42)	Lebanon: Beit Meri (WU: FE, 22.3.2005)	MK027922	MK027784	Mediterranean	perennial	4	tubular	polycolpate	dry	no
A. maximowiczii Kom. (136)	South Korea: near Seoul (CAS, GH: Boufford et al. 25791)	MK027992	MK027864	E Eurasia	perennial	6 or more	tubular	polycolpate	dry	no
A. tinctoria L. (01M_GB24)	Switzerland: Geneva, Bot. Garden (G: A. Natali & J.-F. Manen s.n., Geneva Bot. Garden 780680)	MK028013	N/A	Europe	perennial	6 or more	tubular	polycolpate	dry	no
**sect. *Hexaphylla***										
A. capitata Kit. ex Schult. (51)	Romania: E Carpathians (WU: FE, 9.7.1998)	MK027931	MK027790	Europe	perennial	6 or more	tubular	polycolpate	dry	no
A. hirsuta Desf. (R273M)	Spain (no herbarium voucher)	MK028108	N/A	Mediterranean	perennial	6 or more	tubular	polycolpate	dry	no
A. hirta Ramond (51M_GB20)	Switzerland: Geneva, Bot. Garden (G: A. Natali & J.-F. Manen s.n., Geneva Bot. Garden 814140/0M)	MK028084	N/A	Europe	perennial	6 or more	tubular	polycolpate	dry	no

A. rupestris Tineo (504M)	Italy: Sicilia, Monte Cofano (PAL: L. Robba 12, Oct. 2003)	MK028078	N/A	Mediterranean	perennial	6 or more	tubular	polycolpate	dry	no
**sect. *Thliphthisa***										
A. breviflora Boiss. (41)	Lebanon: Bjarreh (WU: FE, 23.3.2005)	MK027921	MK027783	Mediterranean	perennial	6 or more	tubular	polycolpate	dry	no
A. chlorantha Boiss. & Heldr. (69M_GB23)	Greece: Epirus (WU, G: FE 930413-4401)	MK028092	N/A	Mediterranean	perennial	6 or more	rotate	polycolpate	dry	no
A. cypria Ehrend. (133M)	Cyprus: Ayia-Anna (G: Iter Mediterranea IV 464)	MK028047	N/A	Mediterranean	perennial	6 or more	rotate	polycolpate	dry	no
A. purpurea (L.) Ehrend. (77M_GB19)	Italy: Alpi Apuane (G: A. Natali & J.-F. Manen 011)	MK028096	N/A	Mediterranean	perennial	6 or more	rotate	polycolpate	dry	no
Callipeltis										
C. aperta Boiss. & Buhse (165M)	Israel: northern Negev, Beer Sheva (G, GE: A. Danin et al. It. 15.042)	MK028053	N/A	SW Asia	annual	4	rotate	polycolpate	dry	no
C. cucullaria (L.) DC. (164M)	Spain: Sierra de Gador (G: B. Valdes et al. It. 745/88)	MK028052	N/A							
C. cucullaria (L.) DC. (61)	Jordan: Aljun (WU: FE 1, 10.4.2000)	N/A	MK027797	Mediterranean & SW Asia	annual	4	rotate	polycolpate	dry	no
C. microstegia Boiss. (17)	Iran: Shiraz (WU: FE, 11.4.2006)	MK027898	N/A	SW Asia	annual	4	rotate	polycolpate	dry	no
Crucianella										
C. aegyptiaca L. (131M)	Cyprus: Cape Kiti (G: Iter Mediterranea IV 299)	MK028045	N/A	Mediterranean	annual	6 or more	tubular	polycolpate	dry	no
C. angustifolia L. (29M_GB28)	France: Corsica, Francardo (G: D. Jeanmonod & A. Natali J5044)	MK028068	N/A	Mediterranean	annual	6 or more	tubular	polycolpate	dry	no
C. gilanica Trin. (285M)	Iran: Prov. East Azerbajan, Amirabad (G, GE: K.H. Rechinger 43278)	MK028066	N/A	SW Asia	perennial	6 or more	tubular	polycolpate	dry	no
C. imbricata Boiss. (15)	Greece: Kalimnos (WU: Zervou 2524, 29.4.2002)	MK027896	N/A	Mediterranean	annual	6 or more	tubular	polycolpate	dry	no
C. latifolia L. (16)	Greece: Kalimnos (WU: Zervou 966, 15.4.1999)	MK027897	MK027761	Mediterranean	annual	6 or more	tubular	polycolpate	dry	no

C. macrostachya Boiss. (5)	Lebanon: Byblos (WU: FE, 22.3.2005)	MK027887	MK027751	Mediterranean	annual	6 or more	tubular	polycolpate	dry	no
C. maritima L. (123M)	Italy: Sardinia, Platamona (G: A. Natali 56803)	MK028039	N/A							
C. maritima L. (502M)	Italy: Sicilia, Trapani, Capo Granitola (PAL: L. Robba 11, Oct. 2003)	MK028076	N/A	Mediterranean	perennial	4	tubular	polycolpate	dry	no
C. patula L. (283M)	Spain: Salamanca, Las Calizas del Cabezal Vieijo (G, GE: E. Rico 53)	MK028064	N/A	Mediterranean	annual	4	tubular	polycolpate	dry	no
C. rupestris Guss. (501M)	Italy: Sicilia, Trapani, Capo Granitola (PAL: L. Robba s.n.)	MK028075	N/A	Mediterranean	perennial	6 or more	tubular	polycolpate	dry	no
C. suaveolens C.A. Mey. (284M)	Iran: 40 km E Meshlrins hahz (G, GE: Herbier Henri Pabot 3222)	MK028065	N/A	SW Asia	perennial	4	tubular	polycolpate	dry	no
Cruciata										
C. articulata (L.) Ehrend. (50)	Lebanon: Zahle (WU: FE, 26.3.2005)	MK027930	MK027789	SW Asia	annual	4	rotate	polycolpate	dry	no
C. articulata (L.) Ehrend. (63)	Jordan: Aljun (WU: FE, 10.4.2000)	MK027938	MK027799							
C. laevipes Opiz (34M_GB26)	France: Corsica, Radicale (G: D. Jeanmonod, A. Natali & R. Palese J4198)	MK028072	N/A	Europe	perennial	4	rotate	polycolpate	dry	no
C. pedemontana (Bellardi) Ehrend. (125M)	France: Corsica, Col de Vergio (G: G. Dutartre s.n.)	MK028041	N/A	Mediterranean & SW Asia	annual	4	rotate	polycolpate	dry	no
C. taurica (Pall. ex Willd.) Ehrend. (282M)	Turkey: Kütahya - Gediz (G, GE: M. Nydegger 40743)	MK028063	N/A	SW Asia	perennial	4	rotate	polycolpate	dry	no
C. verna (Scop.) Gutermann & Ehrend. (= *C. glabra* (L.) Opiz) (22M_GB27)	Italy: Elba Island (G: A. Natali & M.-A. Thiébaud N57761 [as “Cruciata glabra”)	MK028059	N/A	Europe	perennial	4	rotate	polycolpate	dry	no
Didymaea										

D. alsinoides (Schltdl. & Cham.) Standl. (71M_GB37)	Costa Rica (WU: M. Kiehn s.n.)	MK028093	N/A	Central America	perennial	2 + 2 small stipules	rotate	polycolpate	fleshy	no
Galium										
**sect. *Aparine***										
G. aparine L. (15M_GB13)	France: Corsica, Pietrabugno (G: D. Jeanmonod & A. Natali s.n.)	MK028051	N/A	Europe & SW Asia	annual	6 or more	rotate	polycolpate	dry	yes
G. monachinii Boiss. & Heldr. (9)	Greece: Kalimnos (WU: Zervou 2614, 28.4.2007)	MK027891	MK027755	Mediterranean	annual	6 or more	rotate	polycolpate	dry	yes
G. pisiferum Boiss. (44)	Lebanon: Saida, Eschmun Tempel (WU: FE, 21.3.2006)	MK027924	N/A							
G. pisiferum Boiss. (45)	Lebanon: Qana (WU: FE, 21.3.2005)	MK027925	MK027786	Mediterranean	annual	6 or more	rotate	polycolpate	dry	no
G. pisiferum Boiss. (46)	Lebanon: Nahr el Kalb (WU: FE, 22.3.2005)	MK027926	N/A							
G. spurium L. (110M)	China: Zhejang (G: C.T. Chen 9403001)	MK028030	N/A	Mediterranean & SW Asia	annual	6 or more	rotate	polycolpate	dry	yes
G. spurium L. (31)	Australia (unknown: J.R. Hosking 2776)	MK027911	MK027774	Mediterranean & SW Asia	annual	6 or more	rotate	polycolpate	dry	yes
**sect. *Aparinoides***										
G. debile Desv. (130)	Australia: New South Wales, Moss Vale (NSW 495299: Kodel & Sainty 260 [as "Galium palustre": Thompson, 2009')	MK027988	MK027859	Mediterranean	perennial	6 or more	rotate	polycolpate	dry	no
G. elongatum C. Presl (06M_GB15)	France: Corsica, St. Florent (G: D. Jeanmonod & A. Natali J4966)	MK028018	N/A	Europe	perennial	6 or more	rotate	polycolpate	dry	no
G. innocuum Miq. (131)	China: Yunnan, Tengchong (WU, CAS: Gaoligong Shan 29775)	MK027989	MK027860	E Eurasia	perennial	6 or more	rotate	polycolpate	dry	no
G. magellanicum Hook. f. (78)	Chile: Torres del Paine (WU: FE 75, 4.2.1998)	MK027948	MK027810	South America	perennial	6 or more	rotate	polycolpate	dry	no
G. magellense Ten. (134)	Italy: Gran Sasso (WU: seed exchange)	N/A	MK027862	Mediterranean	perennial	6 or more	rotate	polycolpate	dry	no

G. palustre L. (14M_GB14)	Switzerland: Geneva (G: A. Natali & J.-F. Manen s.n.)	MK028050	N/A	Europe	perennial	6 or more	rotate	polycolpate	dry	no
**sect. *Aspera***										
G. capitatum Bory & Chaub. (11)	Greece: Kalimnos (WU: Zervou 2624, 29.4.2007)	MK027893	MK027757	Mediterranean	annual	6 or more	rotate	polycolpate	dry	no
G. capitatum Bory & Chaub. (12)	Greece: Kalimnos (WU: Zervou 2463, 6.4.2003)	MK027894	MK027758							
G. divaricatum Lam. (04M_GB17)	France: Corsica, Ajaccio (G: D. Jeanmonod, A. Natali & C. Zellweger J3394)	MK028016	N/A	Mediterranean	annual	6 or more	rotate	polycolpate	dry	no
G. intricatum Margot & Reut. (55M_GB8)	Greece: Ionian Islands (WU, G: FE 930409-2501)	MK028088	N/A	Mediterranean	annual	6 or more	rotate	polycolpate	dry	yes
G. minutulum Jord. (129M)	France: Corsica, Punta Cavallata (G: G. Dutartre, 28.4.1983)	MK028042	N/A	Mediterranean	annual	6 or more	rotate	polycolpate	dry	yes
G. murale (L.) All. (13)	Greece: Kalimnos (WU: Zervou 631, 11.4.1999)	MK027895	MK027759	Mediterranean	annual	6 or more	rotate	polycolpate	dry	yes
G. murale (L.) All. (14)	Greece: Telendos (WU: Zervou 943, 21.2.1999)	N/A	MK027760							
G. murale (L.) All. (54M_GB7)	Greece: Ionian Islands (WU, G: FE 930409-2502)	MK028087	N/A							
G. parisiense L. (10M_GB18)	France: Corsica, Radicale (G: D. Jeanmonod, A. Natali & R. Palese J4186)	MK028029	N/A	Mediterranean	annual	6 or more	rotate	polycolpate	dry	no
G. verticillatum Danthoine ex Lam. (505M)	Italy: Sicilia, Madonie (PAL, GE: L. Robba 5, Oct. 2003)	MK028079	N/A	Mediterranean	annual	6 or more	rotate	polycolpate	dry	no
**sect. *Cymogalia***										
G. paradoxum Maxim. (139)	China: Henan, Neixiang Xian (CAS, GH: Boufford et al. 26275)	MK027995	MK027867	E Eurasia	perennial	2 + 2 enlarged stipules	rotate	polycolpate	dry	yes
G. paradoxum Maxim. (140)	China: Sichuan, Danba Xian (WU, GH: Boufford et al. 38045)	MK027996	MK027868	E Eurasia	perennial	2 + 2 enlarged stipules	rotate	polycolpate	dry	yes
**sect. *Depauperata***										

G. exile Hook. f. (120)	China: Sichuan, Rangtang Xian (WU, GH: Boufford et al. 39294)	MK027978	MK027849	E Eurasia	annual	2 + 2 enlarged stipules	rotate	polycolpate	dry	yes
G. exile Hook. f. (154)	China: Qinghai, Nangqen Xian (CAS: Ho et al. 2532)	N/A	MK027879							
**sect. *Galium***										
G. aetnicum Biv. (21M)	Italy: Sardegna, Capraia Island (G: A. Natali & M.-A. Thiébaud N57944)	MK028058	N/A	Mediterranean	perennial	6 or more	rotate	polycolpate	dry	no
G. album Mill. (03M_GB2)	France: Corsica, Solenzara (G: D. Jeanmonod, A. Natali & R. Palese s.n.)	MK028015	N/A							
G. album Mill. (107)	Bulgaria: Lozenska Mt., 650 m (WU: M. Anchev A00683)	MK027967	MK027838	Europe	perennial	6 or more	rotate	polycolpate	dry	no
G. album Mill. (26M)	France: Corsica, St. Florent (G: D. Jeanmonod & A. Natali J4963)	MK028062	N/A							
G. arenarium Loisel. (93M)	France: Aude, Montdieu (WU: FE 2038)	MK028105	N/A	Mediterranean	perennial	6 or more	rotate	polycolpate	dry	no
G. asparagifolium Boiss. & Heldr. (97M)	Greece: Mt. Parnassos (WU: FE 1966)	MK028107	N/A	Mediterranean	perennial	6 or more	rotate	polycolpate	dry	no
G. capense Thunb. (70)	South Africa: Zederberg (WU: FE 84b, 11.12.1999)	MK027944	N/A							
G. capense Thunb. (71)	South Africa: Ceres-Zederberg (WU: FE 82, 11.12.1999)	MK027945	MK027804	Africa	perennial	6 or more	rotate	polycolpate	dry	no
G. corrudifolium Vill. (31M)	Italy: Elba Island (G: A. Natali & M.-A. Thiébaud N56941)	MK028069	N/A	Mediterranean	perennial	6 or more	rotate	polycolpate	dry	no
G. glaucum L. (507M)	Central Europe (no herbarium voucher)	MK028081	N/A	Europe	perennial	6 or more	tubular	polycolpate	dry	no
G. humifusum M. Bieb. (60)	Jordan: Petra (WU: FE, 7.4.2000 [as “Galium cf. humifusum”])	MK027936	MK027796	Europe & SW Asia	perennial	6 or more	rotate	polycolpate	dry	no

G. laevigatum L. (101)	Italy: Alpi Bergamasche, Passo S. Marco Bema-Schlucht (WU: FE, 16.8.2000)	MK027962	MK027832	Europe	perennial	6 or more	rotate	polycolpate	dry	no
G. libanoticum Ehrend. (47)	Lebanon: Bjarreh (WU: FE)	MK027927	MK027787	Mediterranean	perennial	6 or more	rotate	polycolpate	dry	no
G. lovcense Urum. (109)	Bulgaria: Pirin Mt., 1660 m (WU: M. Anchev A00747-2)	MK027968	MK027839	Mediterranean	perennial	6 or more	rotate	polycolpate	dry	no
G. lucidum All. (08M)	France: Corsica, Strette (G: D. Jeanmonod & A. Natali J4964)	MK028020	N/A	Europe	perennial	6 or more	rotate	polycolpate	dry	no
G. lucidum All. (18M_GB1_201)	Italy: Elba Island (G: A. Natali & M.-A. Thiébaud N56959)	MK028056	N/A							
G. lucidum All. (2)	Bulgaria: loc. 07-78 (WU: V. Vladimirov)	MK027884	MK027748	Europe	perennial	6 or more	rotate	polycolpate	dry	no
G. lucidum All. (57M)	Greece: Ionian Islands (WU: FE 930409-2503)	MK028090	N/A							
G. maritimum L. (94M)	France: Morbihan (WU: FE 2051)	MK028106	N/A	Mediterranean	perennial	6 or more	rotate	polycolpate	dry	no
G. mirum Rech. f. (122M)	Bulgaria: Besaparski (G: Anchev & Mincho A9234)	MK028038	N/A	Mediterranean	perennial	6 or more	rotate	polycolpate	dry	no
G. mollugo L. (12M_122M)	France: Corsica, Calvi (G: D. Jeanmonod, A. Natali & C. Zellweger s.n.)	MK028043	N/A	Europe	perennial	6 or more	rotate	polycolpate	dry	no
G. perralderi Coss. (81)	Tunisia: Sidi Saad, Stausee (WU: FE, 23.4.2001 [as “Galium perrieri”])	MK027949	MK027812	Mediterranean	perennial	6 or more	rotate	polycolpate	dry	no
G. poiretianum Ball (82)	Tunisia: Cap Bon (WU: FE, 16.4.2001)	MK027950	MK027813	Mediterranean	perennial	6 or more	rotate	polycolpate	dry	no
G. rhodopeum Velen. (106)	Bulgaria: Bessaparski hills (WU: M. Anchev A00715)	MK027966	MK027837	Mediterranean	perennial	6 or more	rotate	polycolpate	dry	no
G. rhodopeum Velen. (121M)	Bulgaria: Besaparski (G: Anchev & Mincho A9232)	MK028037	N/A							

G. sylvaticum L. (83M)	Switzerland: Geneva, Versoix (G: A. Natali & J.-F. Manen 018)	MK028102	N/A	Europe	perennial	6 or more	rotate	polycolpate	dry	no
G. thymifolium Boiss. & Heldr. (92M)	Greece: Mt. Parnasse (WU: FE 1989)	MK028104	N/A	Mediterranean	perennial	6 or more	rotate	polycolpate	dry	no
G. tunetanum Lam. (508M)	Italy: Sicilia, Mazzara (PAL, GE: L. Robba 4)	MK028082	N/A	Mediterranean	perennial	6 or more	rotate	polycolpate	dry	no
G. verum L. (1)	Bulgaria: loc. 07-70 G-2 (WU: V. Vladimirov)	MK027883	MK027747	Europe & E Eurasia	perennial	6 or more	rotate	polycolpate	dry	no
G. verum L. (23M)	Switzerland: Geneva, Lullier (G: A. Natali & J.-F. Manen 009)	MK028060	N/A							
**sect. *Hylaea***										
G. echinocarpum Hayata (152)	Taiwan: Chiayi Co., 3400-3700m (PE: Yu-pin Cheng 2391)	MK028007	MK027877	E Eurasia	perennial	6 or more	rotate	polycolpate	dry	yes
G. hoffmeisteri (Klotzsch) Ehrend. & Schönb.-Tem. ex R.R. Mill (116)	China: Yunnan, Lushui Xian (CAS: Gaoligong Shan 10216 [as "Galium bungei"])	MK027974	MK027845							
G. hoffmeisteri (Klotzsch) Ehrend. & Schönb.-Tem. ex R.R. Mill (124)	China: Yunnan (WU, GH: Gaoligong Shan 23338)	MK027982	MK027853							
G. hoffmeisteri (Klotzsch) Ehrend. & Schönb.-Tem. ex R.R. Mill (125)	China: Yunnan (WU, GH: Gaoligong Shan 24112)	MK027983	MK027854	E Eurasia	perennial	6 or more	rotate	polycolpate	dry	yes
G. hoffmeisteri (Klotzsch) Ehrend. & Schönb.-Tem. ex R.R. Mill (126)	China: Yunnan (WU, GH: Gaoligong Shan 29310)	MK027984	MK027855							
G. hoffmeisteri (Klotzsch) Ehrend. & Schönb.-Tem. ex R.R. Mill (127)	China: Yunnan (WU, GH: Gaoligong Shan 34430)	MK027985	MK027856							

G. hoffmeisteri (Klotzsch) Ehrend. & Schönb.-Tem. ex R.R. Mill (128)	China: Gansu, Wen Xian (WU, CAS, GH: Boufford et al. 37617)	MK027986	MK027857							
G. hoffmeisteri (Klotzsch) Ehrend. & Schönb.-Tem. ex R.R. Mill (129)	China: Sichuan, Baiyu Xian (WU, GH: Boufford et al. 37059)	MK027987	MK027858	E Eurasia	perennial	6 or more	rotate	polycolpate	dry	yes
G. japonicum Makino (*G. nipponicum* Makino) (159)	Japan: Hokkaido, Furano (WU: Sohma & Takahashi 708 [as “Galium nipponicum”])	MK028009	N/A	E Eurasia	perennial	6 or more	rotate	polycolpate	dry	yes
G. japonicum Makino (*G. nipponicum* Makino) (160)	Japan: Honshu, Yamanashi Pref. (WU: Flora Jap. Exsiccata 516 [as “Galium triflorum var. nipponicum”])	MK028010	MK027880	E Eurasia	perennial	6 or more	rotate	polycolpate	dry	yes
G. odoratum (L.) Scop. (80M_GB6)	Switzerland: Geneva, Versoix (G: A. Natali & J.-F. Manen 016)	MK028099	N/A	Europe & E Eurasia	perennial	6 or more	tubular	polycolpate	dry	yes
G. odoratum (L.) Scop. (86)	Korea: Villung Island (WU: Tod Stuessy [as “Asperula odorata”])	MK027953	MK027817	Europe & E Eurasia	perennial	6 or more	tubular	polycolpate	dry	yes

**sect. *Jubogalium***										
G. canum Req. ex DC. (49)	Cyprus: Kyrenia (WU: FE, Feb. 2005)	MK027929	MK027788							
G. canum Req. ex DC. (8)	Greece: Kalimnos (WU: Zervou 2624, 29.4.2007)	MK027890	MK027754	Mediterranean	perennial	6 or more	rotate	polycolpate	dry	no
G. graecum L. (6)	Greece: Kalimnos (WU: Zervou 1887, 8.4.2000)	MK027888	MK027752	Mediterranean	perennial	6 or more	rotate	polycolpate	dry	no
G. graecum L. (7)	Greece: Kalimnos (WU: Zervou 2587, 27.4.2006)	MK027889	MK027753							
G. judaicum Boiss. (57)	Jordan: Gerasa (WU: FE A2, 10.4.2000)	MK027935	N/A	Mediterranean	annual	6 or more	rotate	polycolpate	dry	no
G. jungermannioides Boiss. (43)	Lebanon: Balamand (WU: FE 24.3.2005)	MK027923	MK027785	Mediterranean	perennial	6 or more	rotate	polycolpate	dry	no
G. petrae Oliv. ex Hart (59)	Jordan: Petra (WU: FE, 17.4.2000)	N/A	MK027795	Mediterranean	perennial	6 or more	rotate	polycolpate	dry	no
G. setaceum subsp. *decaisnei* (Boiss.) Ehrend. (3)	Iran: Shiraz (WU: FE, 11.4.2006)	MK027885	MK027749	Mediterranean	annual	6 or more	rotate	polycolpate	dry	no
**sect. *Kolgyda***										
G. ceratopodum Boiss. (19)	Iran: Pasagarde (WU: FE, 13.4.2006)	MK027899	MK027762	SW Asia	annual	6 or more	rotate	polycolpate	dry	no
G. tricornutum Dandy (10)	Greece: Kalimnos (WU: Zervou 2679, 12.4.2007)	MK027892	MK027756	Mediterranean	annual	6 or more	rotate	polycolpate	dry	no
G. tricornutum Dandy (76M)	Switzerland: Geneva, Bot. Garden (G: A. Natali & J.-F. Manen 014 - Geneva Bot. Garden)	MK028095	N/A							
G. verrucosum Huds. (24M_GB11)	France: Corsica, Bonifacio (G: D. Jeanmonod, A. Natali & D. Roguet J3980)	MK028061	N/A							
G. verrucosum Huds. (4)	Cyprus: Paphos (WU: FE, Feb. 2005)	MK027886	MK027750	Mediterranean	annual	6 or more	rotate	polycolpate	dry	no
G. verrucosum Huds. (48)	Cyprus: Kowrion (WU: FE, Feb. 2005)	MK027928	N/A							
G. verrucosum Huds. (56M)	Greece: Ionian Islands (WU: FE 930409-25)	MK028089	N/A							

**sect. *Leptogalium***										
G. × *centroniae* Cariot (94)	Italy: Alpi Bergamasche – S. Marco Pan ob Alboredo, ca. 1000 m (WU: FE, 16.8.2000)	N/A	MK027825	Europe	perennial	6 or more	rotate	polycolpate	dry	no
G. anisophyllon Vill. (100)	Switzerland: Splügen (WU: FE, 12.8.2000)	N/A	MK027831							
G. anisophyllon Vill. (103)	Italy: eastern Alpi Bergamasche: - Mt. Cocco, ca. 2400 m (WU: P. Schönswetter, 16.8.2000)	N/A	MK027834							
G. anisophyllon Vill. (58)	Switzerland: Pilatus (WU: FE, 3.6.2001)	N/A	MK027794							
G. anisophyllon Vill. (85)	Austria: Niederösterreich, Rax, Bergstation Seilbahn – Ottohaus, ca. 1600 m (WU: FE, 30.6.2002)	N/A	MK027816	Europe	perennial	6 or more	rotate	polycolpate	dry	no
G. anisophyllon Vill. (87)	Romania, Carpathians, Hagimas (WU: FE, July 1998)	N/A	MK027818							
G. anisophyllon Vill. (97)	Italy: Alpi Bergamasche, Passo di S.Marco ob Morbegno, c.2000 m (WU: FE, 15.8.2000)	N/A	MK027828							
G. anisophyllon Vill. (98)	Austria: Kärnten, Hoch-Obir, 2100 m (WU: FE, 21.7.1998)	N/A	MK027829							
G. corsicum Spreng. (09M_GB16)	France: Corsica, Col St. Jean (G: D. Jeanmonod & A. Natali J4931)	MK028021	N/A	Mediterranean	perennial	6 or more	rotate	polycolpate	dry	no
G. noricum Ehrend. (77)	Austria: Kärnten, Hochobir, ca. 2100 m (WU: FE, 21.7.1998)	N/A	MK027809	Europe	perennial	6 or more	rotate	polycolpate	dry	no
G. noricum Ehrend. (79)	Austria: Kärnten, Petzen, ca. 1950 m (WU: FE, 23.7.2001)	N/A	MK027811							
G. valentinum Lange (89)	Spain: Valencia (WU: M. Boşcaiu)	N/A	MK027820	Mediterranean	perennial	6 or more	rotate	polycolpate	dry	no

**sect. *Platygalium***										
G. araucanum Phil. (76)	Chile: Pucón (WU: P. Menzel & M.Kiehn 971105-4/1)	MK027947	MK027808	South America	perennial	4	rotate	polycolpate	dry	no
G. araucanum Phil. (92)	Chile: Nahuelbuta (WU: FE, Jan.1998)	MK027957	MK027823							
G. baillonii Brandza (52M_GB10)	Romania: Prov. Arges (WU, G: FE 890821-3001)	MK028085	N/A	Europe	perennial	4	rotate	polycolpate	dry	no
G. bermudense L. (287M)	USA: Georgia, Sapelo Island (G, GE: W.H. Duncan 20648)	MK028067	N/A	North America	perennial	4	rotate	polycolpate	fleshy	no
G. bigeminum Griseb. (62)	Argentina: Cordoba (WU: FE 108, 18.2.1998)	MK027937	MK027798	South America	perennial	4	rotate	polycolpate	fleshy	no
G. boreale L. (79M_GB9_209)	Switzerland: Geneva, Bot. Garden (G: A. Natali & J.-F. Manen s.n., Geneva Bot. Garden 814159/0)	MK028098	N/A							
G. boreale L. (R286M)	Kashmir, Chhachos Pass (G: L.G. Webster & E. Nasir 6405)	MK028113	N/A	Europe & E Eurasia	perennial	4	rotate	polycolpate	dry	no
G. bungei Steud. (102M)	Japan: Nikko (WU, G: FE & HB 930825-0101)	MK028024	N/A							
G. bungei Steud. (123)	China: Sichuan, Dujiangyan Xian (CAS, GH: Bouford et al. 24129 [as “Galium aff. paradoxum”])	MK027981	MK027852							
G. bungei Steud. (34)	China: Beijing, Great Wall (WU: FE, 14.8.2006)	MK027914	MK027777	E Eurasia	perennial	4	rotate	polycolpate	dry	yes
G. bungei Steud./*G. yunnanense* H. Hara & C.Y. Wu (148)	China: Yunnan (CAS, WU: Gaoligong Shan 17673)	MK028004	MK027875	E Eurasia	perennial	4	rotate	polycolpate	dry	yes
G. californicum Hook. & Arn. (111M)	USA: California, Orinda (G: Dempster s.n.)	MK028031	N/A	North America	perennial	4	rotate	polycolpate	dry	no
G. californicum Hook. & Arn. (66)	USA: California, Monterey (WU: FE 31, 25.7.1999)	MK027940	N/A							

G. ciliare Hook. f. (29)	Australia: Tasmania, Cradle Valley (NSW 744321: B.G. Briggs 9783, 23.2.2007)	MK027909	MK027772	Australia	perennial	4	rotate	polycolpate	dry	no
G. ciliare Hook. f. (30)	Australia: NSW, Southern Tablelands, Kosciusko (NSW 744306: B.G. Briggs 9769, 9.2.2007 [as "Asperula"])	MK027910	MK027773							
G. circaezans Michx. (20)	USA: West Virginia, Shenandoah Mts. (WU: FE, 8.5.2007)	MK027900	MK027763	North America	perennial	4	rotate	polycolpate	dry	yes
G. elegans Wall. (104)	China: Yunnan (a) (WU: Schneeweiss)	MK027964	MK027835							
G. elegans Wall. (105)	China: Yunnan (b) (WU: Schneeweiss)	MK027965	MK027836	E Eurasia	perennial	4	rotate	polycolpate	dry	yes
G. elegans Wall. (135)	China: Yunnan, Longling Xian (CAS: Gaoligong Shan 23691)	MK027991	MK027863							
G. elegans Wall. (153)	China: Yunnan, Fugong Xian (WU, CAS: Gaoligong Shan 28433)	MK028008	MK027878							
G. elegans Wall. (38)	China: Kunming (WU: FE, 27.7.2006)	MK027918	MK027780	E Eurasia	perennial	4	rotate	polycolpate	dry	yes
G. eriocarpum Bartl. ex DC. (74)	Chile: Santiago: Andes (WU: FE 3, 17.1.1998 [as "Galium eriophorum"])	N/A	MK027807	South America	perennial	4	rotate	polycolpate	dry	no
G. gaudichaudii DC. (28)	Australia: NSW, Blackfellows Knob (NSW, WU: J.R. Hosking 2778)	MK027908	MK027771	Australia	perennial	4	rotate	polycolpate	dry	no
G. glandulosum Hand.-Mazz. (122)	China: Sichuan, Yajiang Xian (WU, GH: Boufford et al. 35962)	MK027980	MK027851							
G. glandulosum Hand.-Mazz. (121)	China: Sichuan, Derong Xian (WU, GH: Boufford et al. 30811)	MK027979	MK027850	E Eurasia	perennial	4	rotate	polycolpate	dry	yes
G. grande McClatchie (112M)	USA: California, Chantry Flat (G: Dempster & Stebbins 3975)	MK028032	N/A	North America	perennial	4	rotate	polycolpate	dry	no

G. hirtum Lam. (56)	Brazil: Porto Allegre (WU: FE B1, 6.3.1998)	MK027934	MK027793	South America	perennial	4	rotate	polycolpate	fleshy	no
G. humile Cham. & Schltdl. (55)	Brazil: Haimbezinho (WU: FE B2, 7.3.1998 [as "Relbunium humile"])	N/A	MK027792	South America	annual	4	rotate	polycolpate	dry	no
G. hypocarpium (L.) Endl. ex Griseb. (113M)	Costa Rica (WU: Vienna Bot. Garden HBV RR 92/2)	MK028033	N/A	South America	perennial	4	rotate	polycolpate	fleshy	no
G. kamtschaticum Steller ex Schult. & Schult. f. (101M)	Japan: Nikko Nat. Park (WU, G: FE 930826-0500)	MK028023	N/A	E Eurasia	perennial	4	rotate	polycolpate	dry	yes
G. kinuta Nakai & H. Hara (106M)	Japan: Nikko (WU: FE 930827-0102)	MK028026	N/A	E Eurasia	perennial	4	rotate	polycolpate	dry	no
G. linearifolium Turcz. (133)	China: Beijing (CAS: Changping Exp. 3, 15-6-2000)	MK027990	MK027861	E Eurasia	perennial	4	rotate	polycolpate	dry	no
G. migrans Ehrend. & McGill. (120M)	Australia: Cape Le Grand (G: S. Pignatti 719)	MK028036	N/A	Australia	perennial	4	rotate	polycolpate	dry	no
G. multiflorum Kellogg (65)	USA: California, S Topaz Lake (WU: FE 52, 28.7.1999)	MK027939	MK027800	North America	perennial	4	rotate	polycolpate	dry	no
G. richardianum (Gillies ex Hook. & Arn.) Endl. ex Walp. (93)	Argentina: Cordoba (WU: FE 107, 18.2.1998 [as "*Relbunium richardianum*"])	MK027958	MK027824	South America	perennial	4	rotate	polycolpate	dry	no
G. rotundifolium L. (17M)	France: Corsica, Loreto di Casinca (G: D. Jeanmonod & A. Natali J4979)	MK028055	N/A	Europe	perennial	4	rotate	polycolpate	dry	yes
G. rubioides L. (75M_GB4)	Switzerland: Geneva, Bot. Garden (G: A. Natali & J.-F. Manen 013, Geneva Bot. Garden)	MK028094	N/A	Europe	perennial	4	rotate	polycolpate	dry	no
G. scabrum L. (07M_GB12)	France: Corsica, Porto (G: D. Jeanmonod & D. Roguet J4961)	MK028019	N/A	Mediterranean	perennial	4	rotate	polycolpate	dry	yes
G. septentrionale Roem. & Schult. (45M_GB3)	USA: Colorado (G: A. Natali & J.-F. Manen 016)	MK028074	N/A	North America	perennial	4	rotate	polycolpate	dry	no

G. sparsiflorum W. Wight (67)	USA: California, Mariposa (WU: FE 40, 26.7.1999 [as "grande 4-na"])	MK027941	MK027801	North America	perennial	4	rotate	polycolpate	fleshy	no
G. trichocarpum DC. (73)	Chile: Tiltil (WU: FE 12, 19.1.1998 [as "Galium lateramosum"])	MK027946	MK027806	South America	perennial	4	rotate	polycolpate	dry	no
G. yunnanense H. Hara & C.Y. Wu (146)	China: Yunnan, Gongshan (WU, CAS: Gaoligong Shan 33243)	MK028002	N/A							
G. yunnanense H. Hara & C.Y. Wu (147)	China: Yunnan, Songming Xian (WU, GH: Boufford et al. 34983)	MK028003	MK027874	E Eurasia	perennial	4	rotate	polycolpate	dry	yes
**sect. *Trachygalium***										
G. acutum Edgew. (150)	China: Sichuan, Dujianyan (WU, GH: Boufford et al., 24796)	MK028006	N/A	E Eurasia	perennial	6 or more	rotate	polycolpate	dry	no
G. asperifolium Wall. (111)	China: Yunnan, Fugong Xian (WU, CAS: Gaoligong Shan 27567 [as "Galium asperuloides"])	MK027969	MK027840							
G. asperifolium Wall. (112)	China: Yunnan, Gongshan (WU, CAS: Gaoligong Shan 33268 [as "Galium asperuloides"])	MK027970	MK027841							
G. asperifolium Wall. (113)	China: Sichuan, Yajiang Xian (WU, GH: Boufford et al. 35940 [as "Galium sikkimense"])	MK027971	MK027842	E Eurasia	perennial	6 or more	rotate	polycolpate	dry	no
G. asperifolium Wall. (114)	China: Xizang (Tibet), Jiangda Xian (WU, GH: Boufford et al. 31222)	MK027972	MK027843							
G. asperifolium Wall. (36)	China: Kunming (WU: FE, 27.7.2006)	MK027916	MK027779	E Eurasia	perennial	6 or more	rotate	polycolpate	dry	no
G. blinii H. Lév. (115)	China: Yunnan, Fugong Xian (WU, CAS: Gaoligong Shan 26993)	MK027973	MK027844	E Eurasia	perennial	6 or more	rotate	polycolpate	dry	no

G. blinii H. Lév. (37)	China: Lake Bita (WU: FE, 02.8.2006 [as "Galium luisae"])	MK027917	N/A	E Eurasia	perennial	6 or more	rotate	polycolpate	dry	no
G. dahuricum Turcz. ex Ledeb. (118)	China: Sichuan, Nanchuan Co. (CAS: Liu Zheng-yu 17748)	MK027976	MK027847	E Eurasia	perennial	6 or more	rotate	polycolpate	dry	no
G. dahuricum Turcz. ex Ledeb. (33)	China: Shaanxi, Taibai Mts. (WU: FE, 19.8.2006)	MK027913	MK027776	E Eurasia	perennial	6 or more	rotate	polycolpate	dry	no
G. glabriusculum Ehrend. (*G. smithii* Cuf.) (144)	China: Xizang (Tibet), Jiangda Xian (WU, GH: Bouford et al. 31706)	MK028000	MK027872	E Eurasia	perennial	6 or more	rotate	polycolpate	dry	yes
G. megacyttarion R.R. Mill (137)	China: Sichuan (WU: FE 27161)	MK027993	MK027865	E Eurasia	perennial	6 or more	rotate	polycolpate	dry	no
G. mexicanum subsp. *asperrimum* (A.Gray) Dempster (68)	USA: Mariposa: Midpine (WU: FE 35, 26.7.1999 [as "Galium asperrimum"])	MK027942	MK027802	North America	perennial	6 or more	rotate	polycolpate	dry	no
G. prattii Cufod. (142)	China: Sichuan, Shimian Xian (WU: Boufford et al. 32812)	MK027998	MK027870	E Eurasia	perennial	6 or more	rotate	polycolpate	dry	no
G. prattii Cufod./*G. blinii* H. Lév. (141)	China: Sichuan, Luding Xian (WU, GH: Boufford et al. 27309)	MK027997	MK027869	E Eurasia	perennial	6 or more	rotate	polycolpate	dry	no
G. pseudoasprellum Makino (100M_250)	Japan: Mt. Fuji (WU, G: FE 930910-3801)	MK028022	N/A							
G. pseudoasprellum Makino (108M)	Japan, Hakuba (WU: FE 930904-1501)	MK028027	N/A							
G. pseudoasprellum Makino (117)	China: Qinghai, Nanqen Xian (PE, CAS, E: Ho et al. 2535 [as "Galium dahuricum var. lasiocarpum"])	MK027975	MK027846							
G. pseudoasprellum Makino (119)	Russia: Vladivostok (PE: Pl. Vasc. Or. Extr. Ross. Exsiccata 614 [as "Galium dahuricum var. lasiocarpum"])	MK027977	MK027848	E Eurasia	perennial	6 or more	rotate	polycolpate	dry	yes

G. pseudoasprellum Makino (35)	China: Taibai Mt., Red River valley (WU: FE, 18.8.2006 [as "Galium dahuricum var. lasiocarpum"])	MK027915	MK027778	E Eurasia	perennial	6 or more	rotate	polycolpate	dry	yes
G. pseudoasprellum Makino (39)	China: Zhongdian (WU: FE, 05.8.2006 [as "Galium dahuricum var. lasiocarpum"])	MK027919	MK027781	E Eurasia	perennial	6 or more	rotate	polycolpate	dry	yes
G. rebae R.R. Mill (143)	China: Yunnan, Gongshan Xian (CAS: Gaoligong Shan 16976, 4.10.2002)	MK027999	MK027871	E Eurasia	perennial	6 or more	rotate	polycolpate	dry	no
G. rivale (Sm.) Griseb. (52)	Slowakei (WU: FE, 16.6.2002)	MK027932	N/A	Europe	perennial	6 or more	tubular	polycolpate	dry	no
G. sungpanense Cufod. (145)	China: Sichuan, Baiyu Xian (WU, GH: Boufford et al. 37009)	MK028001	MK027873	E Eurasia	perennial	6 or more	rotate	polycolpate	dry	yes
G. triflorum Michx. (21)	USA: West Virginia, Shenandoah Mts. (WU: FE, 8.5.2007)	MK027901	MK027764	E Eurasia & North America	perennial	6 or more	rotate	polycolpate	dry	yes
G. triflorum Michx. (69)	USA: California, Coast Ranges S of San Francisco (WU: FE 3, 22.7.1999)	MK027943	MK027803							
G. uliginosum L. (53)	Slowakei (WU: FE, 16.6.2002)	MK027933	MK027791							
G. uliginosum L. (96)	Austria: Kärnten, St. Georgen (WU: FE, 25.7.1998)	MK027960	MK027827							
G. uliginosum L. (99)	Austria: Kärnten, St. Georgen (WU: FE, 25.7.1998)	MK027961	MK027830	Europe	perennial	6 or more	rotate	polycolpate	dry	no
Kelloggia										
K. chinensis Franch. (149)	China: Sichuan, Xiangcheng Xian (GH, CAS: Boufford et al. 28360)	MK028005	MK027876	E Eurasia	perennial	2 + 2 small stipules	tubular	3-colpate	dry	yes
K. chinensis Franch. (GB34)	China: Yunnan, Zhongdian, Napaihai (KUN:)	AY570765	N/A							

K. chinensis Franch. (GB35)	China: Xizang, Mangkang (F:)	AY570764	N/A							
K. galioides Torr. (GB32_8865)	USA: California, Warner Mt. (CAS, KUN)	AY570768	N/A	North America	perennial	2 + 2 small stipules	tubular	3-colpate	dry	yes
K. galioides Torr. (GB33)	USA: California, Modoc Mt. (CAS, KUN)	AY570766	N/A							
Mericarpaea										
M. ciliata (Banks & Sol.) Eig (166M)	Israel: Samarian Desert (G: A. Danin et al. It. 08062 [as “Mericarpaea vaillantioides”])	MK028054	N/A	SW Asia	annual	6 or more	rotate	polycolpate	dry	yes

Phuopsis										
P. stylosa (Trin.) Benth. & Hook. f. ex B.D. Jacks. (35M_GB38)	Switzerland: Geneva, Bot. Garden (G: A. Natali & J.-F. Manen s.n., Geneva Bot. Garden 916798)	MK028073	N/A	SW Asia	perennial	6 or more	tubular	polycolpate	dry	no
Rubia										
**sect. *Oligoneura***										
R. cordifolia L. (103M)	Japan: Nikko (WU, G: F. Erhrendorfer & HB 930825-0101)	MK028025	N/A							
R. cordifolia L. (109M)	China: South China Bot. Garden Gouangzhou (CAS, WU: C.T. Chen 9404002)	MK028028	N/A	E Eurasia & Africa	perennial	4_6 or more	rotate	polycolpate	fleshy	no
R. cordifolia L. (87M)	Tanzania (WU, G: C. Puff 860816-1/16)	MK028103	N/A							
R. pseudogalium Ehrend. (163)	China: Yunnan, Bawan Xian (CAS, WU: Gaoligong Shan 23837)	MK028011	MK027881	E Eurasia	perennial	4	rotate	polycolpate	fleshy	no
R. pseudogalium Ehrend. (164)	China: Yunnan, Tengchong (CAS, WU: Gaoligong Shan 29209)	MK028012	MK027882							
R. yunnanensis Diels (138)	China: Yunnan, Longyang Qu (CAS: Gaoligong Shan 18294)	MK027994	MK027866	E Eurasia	perennial	4_6 or more	rotate	polycolpate	fleshy	no
**sect. *Rubia***										
R. danaensis Danin (72)	Jordan: Shobak (WU: FE, 16.4.2000)	N/A	MK027805	Mediterranean	perennial	6 or more	rotate	polycolpate	fleshy	no
R. laurae (Holmboe) Airy Shaw (134M)	Cyprus: Nata-Axilou (G: Iter Mediterranea IV 1043)	MK028048	N/A	Mediterranean	perennial	4	rotate	polycolpate	fleshy	no
R. peregrina L. (33M_GB30)	Italy: Elba Island (G: A. Natali & M.-A. Thiébaud 56965)	MK028071	N/A	Mediterranean	perennial	6 or more	rotate	polycolpate	fleshy	no
R. tenuifolia d'Urv. (40)	Lebanon: Beit Meri (WU: FE, 22.3.2005)	MK027920	MK027782	Mediterranean	perennial	6 or more	rotate	polycolpate	fleshy	no
R. tenuifolia d'Urv. (130M)	Cyprus: Cape Kiti (G: Iter Mediterranea IV 295)	MK028044	N/A							

R. tinctorum L. (11M_GB29)	Switzerland: Geneva, Bot. Garden (G: A. Natali & J.-F. Manen s.n., Geneva Bot. Garden 916690)	MK028035	N/A	SW Asia	perennial	6 or more	rotate	polycolpate	fleshy	no
Sherardia										
S. arvensis L. (02M_GB31)	France: Corsica, St Petrone (G: D. Jeanmonod & A. Natali J5048)	MK028014	N/A	Mediterranean	annual	6 or more	tubular	polycolpate	dry	no
S. arvensis L. (05M)	Switzerland: Geneva, Bot. Garden (G: A. Natali & J.-F. Manen 007, Geneva Bot. Garden)	MK028017	N/A	Mediterranean	annual	6 or more	tubular	polycolpate	dry	no
Valantia										
V. aprica (Sibth. & Sm.) Boiss. & Heldr. (R281M)	Greece: Sterea Ellas, Parnassos, Fterolalka (G, GE: A. Charpin 22.218)	MK028112	N/A	Mediterranean	perennial	4	rotate	polycolpate	dry	no
V. calva Brullo (R280M)	Italy: Sicilia, Linosa, Monte Vulcano (G, GE: S. Brullo s.n., 20.4.1977)	MK028111	N/A	Mediterranean	annual	4	rotate	polycolpate	dry	no
V. deltoidea Brullo (503M_R503M)	Italy: Sicilia, Palermo, Rocca Busambra (PAL: L. Robba 7)	MK028077	N/A	Mediterranean	annual	4	rotate	polycolpate	dry	no
V. hispida L. (132M)	Cyprus: Amathus (G: Iter Mediterranea IV 314)	MK028046	N/A	Mediterranean	annual	4	rotate	polycolpate	dry	no
V. muralis L. (32M_GB39)	France: Corsica, Pigno (G: D. Jeanmonod & A. Natali s.n.)	MK028070	N/A	Mediterranean	annual	4	rotate	polycolpate	dry	no

[a]FE = F. Ehrendorfer
